# Secondary Metabolites from the Marine-Derived Fungus *Dichotomomyces sp*. L-8 and Their Cytotoxic Activity

**DOI:** 10.3390/molecules22030444

**Published:** 2017-03-11

**Authors:** Li-Hong Huang, Yan-Xiu Chen, Jian-Chen Yu, Jie Yuan, Hou-Jin Li, Wen-Zhe Ma, Ramida Watanapokasin, Kun-Chao Hu, Shah Iram Niaz, De-Po Yang, Wen-Jian Lan

**Affiliations:** 1 School of Pharmaceutical Sciences, Sun Yat-sen University, Guangzhou 510006, China; huanglh23@mail2.sysu.edu.cn (L.-H.H.); chenyx239@mail2.sysu.edu.cn (Y.-X C.); Hukch@mail2.sysu.edu.cn (K.-C.H.); lssydp@mail.sysu.edu.cn (D.-P.Y.); 2 Guangdong Technology Research Center for Advanced Chinese Medicine, Guangzhou 510006, China; 3 Zhongshan School of Medicine, Sun Yat-sen University, 74 Zhongshan Road II, Guangzhou 510080, China; lisana121406@163.com (J.-C.Y.); yuanjie@mail.sysu.edu.cn (J.Y.); 4 School of Chemistry, Sun Yat-sen University, Guangzhou 510275, China; ceslhj@mail.sysu.edu.cn; 5 State Key Laboratory of Quality Research in Chinese Medicine, Macau Institute for Applied Research in Medicine and Health, Macau University of Science and Technology, Avenida Wai Long, Taipa 519020, Macau (SAR), China; wzma@must.edu.mo; 6 Department of Biochemistry, Faculty of Medicine, Srinakharinwirot University, Bangkok 10110, Thailand; ramidawa@yahoo.com; 7 Institute of Chemical Sciences, Gomal University, D.I.Khan 29050, Pakistan; shah_iram2000@Yahoo.com

**Keywords:** *Dichotomomyces sp*., secondary metabolites, structure elucidation, cytotoxic activity

## Abstract

Bioassay-guided isolation of the secondary metabolites from the fungus *Dichotomomyces sp*. L-8 associated with the soft coral *Lobophytum crassum* led to the discovery of two new compounds, dichotones A and B (**1** and **2**), together with four known compounds including dichotocejpin C (**3**), bis-*N*-norgliovictin (**4**), bassiatin (**5**) and (3*R*,6*R*)-bassiatin (**6**). The structures of these compounds were determined by 1D, 2D NMR and mass spectrometry. (3*R*,6*R*)*-*bassiatin (**6**) displayed significant cytotoxic activities against the human breast cancer cell line MDA-MB-435 and the human lung cancer cell line Calu3 with IC_50_ values of 7.34 ± 0.20 and 14.54 ± 0.01 μM, respectively, while bassiatin (**5**), the diastereomer of compound **6**, was not cytotoxic.

## 1. Introduction

Coral reef is one of the biggest marine ecosystems. Because corals lack efficient physical protection in the heavily competitive environment, they mainly rely on chemical protection to survive. For the past two decades, it was found that a considerable part of the bioactive substances isolated from marine flora and fauna were actually generated from their symbiotic microorganisms [1]. These findings attracted a great deal of attention in investigating the secondary metabolites from the microorganisms associated with algae and invertebrates, such as soft corals and sponges [2,3]. To date, a number of compounds including polyketides [4], alkaloids [5], lipids [6], anthraquinones [7], terpenes [8] and peptides [6] have been discovered from these commensal microorganisms. These compounds showed wide-ranging biological properties such as anti-fungal, cytotoxic, anti-feedant and anti-viral activities. For example, Dehai Li et al. obtained two unique fumiquinazoline alkaloids with anti-influenza virus A from a mangrove-derived fungus, *Neosartorya udagawae* [9]. 

To explore bioactive natural products, we have initiated a program to screen and discover unique molecules with cytotoxic activity from the marine-derived fungi related to invertebrates, such as soft corals and sea stars. In a previous study, the marine fungus *Neosartorya pseudofischeri* manufactured a series of potent cytotoxic molecules, such as gliotoxin, acetylgliotoxin, and reduced gliotoxin [10]. The fungal strain *Pseudallescheria boydii* produced diverse chemical structures, and some of them showed notable cytotoxic activity against Sf9 cells from the fall armyworm *Spodoptera frugiperda*. In a small-scale culture of *Dichotomomyces sp*., we found that the EtOAc extract of the culture broth showed significant cytotoxic activity with an inhibition rate of 80% at 30 μg/mL against the human breast cancer cell line MDA-MB-435. A literature search revealed that the secondary metabolites of the genus *Dichotomomyces* were abundant and displayed potent bioactivity. For instance, Henrik et al. obtained several indoloditerpenes with antagonistic activity that targeted GPR18 and cannabinoid receptors from *Dichotomomyces cejpii* [11]. Aiming to find novel chemicals with cytotoxic activity, we explored the secondary metabolites of *Dichotomomyces sp*. and obtained two new molecules, dichotones A and B (**1** and **2**), together with four known compounds, dichotocejpin C (**3**), bis-*N*-norgliovictin (**4**), bassiatin (**5**) and (3*R*,6*R*)-bassiatin (**6**). Compound **6** displayed significant cytotoxicity against the human breast cancer cell line MDA-MB-435 and the human lung cancer cell line Calu3. Herein, we report the isolation, structure determination and bioactive evaluation of these compounds.

## 2. Results and Discussion

**Figure 1:**
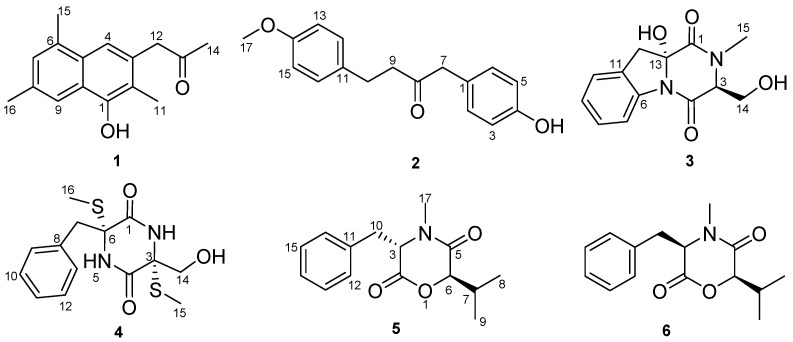
Chemical structures of compounds **1**–**6**.

**Figure 2:**
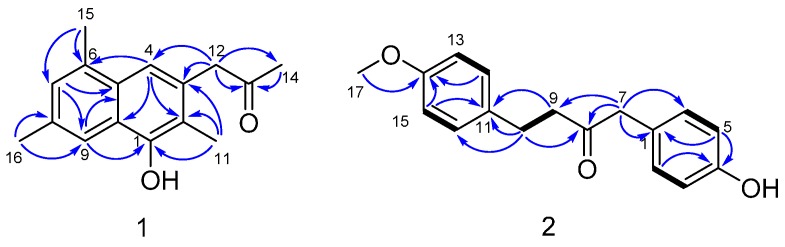
^1^H-^1^H COSY (bold line) and the key HMBC (blue arrow) correlations of compounds **1** and **2**.

Dichotone A (**1**) was obtained as a white amorphous powder. The HRESIMS (high resolution electrospray ionization mass spectroscopy) spectrum showed a strong quasi-molecular ion peak at *m*/*z* 241.1237 [M − H]^−^ (calcd. 241.1234), corresponding to the molecular formula C_16_H_18_O_2_, requiring eight double equivalents. The ^1^H-NMR spectrum of dichotone A (**1**) (Table 1) displayed resonances for 18 protons, including four methyls (δ_H_ 2.16, 2.34, 2.41 and 2.50, each s), one methylene (δ_H_ 3.87, s), three aromatic proton signals (δ_H_ 7.39, 7.49 and 7.61, each s), and one exchangeable singlet proton signal at δ_H_ 4.87 for the hydroxyl moiety. The ^13^C NMR and DEPT spectra (Table 1) showed signals for 16 carbons, including four methyls at δ_C_ 10.8, 17.1, 19.9 and 29.4, one methylene at δ_C_ 50.1, 10 aromatic carbons at δ_C_ 149.9, 132.0, 131.7, 131.5, 128.8, 128.6, 126.2, 125.6, 124.6 and 114.1, and one carbonyl carbon at δ_C_ 207.2. The existence of a naphthalene was deduced by the HMBC correlations (Figure 2) from H-4 to C-2, C-10 and C-6, from H-7 to C-5 and C-9, and from H-9 to C-1 and C-5. Three methyls were substituted at C-2, C-6 and C-8, respectively, which was supported by the HMBC correlations from H_3_-11 to C-1, C-2 and C-3, from H_3_-15 to C-6 and C-7, and from H_3_-16 to C-8 and C-9. The HMBC correlations from H_3_-14 to C-13, from H_2_-12 to C-13, C-14, C-3 and C-4 illustrated that a propan-2-one was linked to C-3. As showed in the HRESIMS spectrum, there was a hydroxyl substituted at C-3. The typical down-field shift of C-1 (δ_C_ 149.9) elaborated the substitution of a remaining hydroxyl at C-1. Thus, the structure of dichotone A (**1**) was constructed as shown in Figure 1.

Dichotone B (**2**) was acquired as a white amorphous powder. It had the molecular formula C_17_H_18_O_3_, which was established on the basis of the HRESIMS ion detected at *m*/*z* 269.1176 [M − H]^−^ (calcd. 269.1183). Analysis of the 1D NMR data and HSQC spectrum suggested the existence of 18 proton and 17 carbon signals, including one keto-carbonyl (δ_C_ 208.3), four couples of symmetric aromatic sp^2^ methine groups (δ_C_ 114.0, δ_H_ 6.79 (d, 8.8); δ_C_ 115.7, δ_H_ 6.77 (d, 8.4); δ_C_ 129.4, δ_H_ 7.04 (d, 8.8); δ_C_ 130.8, δ_H_ 7.02 (d, 9.2) ), four sp^2^ quaternary carbon atoms (δ_C_ 126.4, 133.1, 154.7, 158.0), three sp^3^ methylene groups (δ_C_ 29.1, δ_H_ 2.80 (td, 6.8, 1.6); δ_C_ 43.7, δ_H_ 2.71 (td, 6.8, 1.6); δ_C_ 49.6, δ_H_ 3.57 (s)), one methoxy group (δ_C_ 55.4, δ_H_ 3.77 (s)), and one exchangeable singlet proton (δ_H_ 4.83, brs). Considering the nine degrees of unsaturation calculated from the molecular formula, there were two rings existing in the structure. The presence of two *para*-benzene systems in **2** was deduced from characteristic ^13^C-NMR resonances (Table 1; C-1 to C-6 and C-11 to C-16), along with the HMBC correlations from H-2 (H-6) to C-4, from H-5 (H-3) to C-1 and C-4, from H-15 (H-13) to C-11 and C-14, and from H-12 (H-16) to C-14 and the cross-peaks H-2/H-3 and H-12/H-13 in the COSY spectrum. The HMBC correlations from H_2_-7 to C-8 and C-9, H_2_-9 to C-8, H_2_-10 to C-8 and the COSY correlations between H_2_-9 and H_2_-10 suggested the existence of a 1,4-disubstituted butan-2-one. Two *para*-benzene systems were linked by the 1,4-disubstituted butan-2-one based on the HMBC correlations from H_2_-7 to C-1 and C-6 (C-2), from H_2_-9 to C-11, and from H_2_-10 to C-11 and C-12 (C-16). A methoxy group was linked to C-14 by the evidence of the HMBC correlation from H_3_-17 to C-14. A hydroxyl was substituted at C-4 because of the characteristic down-field shift of C-4 (δ_C_ 157.4), which was in accordance with the molecular formula C_17_H_18_O_3_. Finally, the structure of **2** was established as shown in Figure 1. More spectral data see Supplementary.

Compounds **3**–**6** were identified as dichotocejpin C [12], bis-*N*-norgliovictin [10], bassiatin [13] and (3*R*,6*R*)-bassiatin [14], respectively, by comparing the data with the literature values. 

The (3*R*,6*R*)-bassiatin (**6**) and bassiatin (**5**) are a pair of diastereomers. In the previous research, bassiatin (**5**) was a platelet aggregation inhibitor [13], while (3*R*,6*R*)-bassiatin (**6**) could inhibit proliferation and induce apoptosis of several human cancer cells [15,16,17]. Here, they were screened for their cytotoxic activities against the human breast cancer cell line MDA-MB-435 and the human lung cancer cell line Calu3. Interestingly, their cytotoxic activities were totally different. Compound **6** exhibited distinguished cytotoxic activities against the cell lines MDA-MB-435 and Calu3 with IC_50_ values of 7.34 ± 0.20 and 14.54 ± 0.01 μM, respectively. However, compound **5** did not show any distinct activity with IC_50_ values higher than 50μM. The cytotoxic activities of compounds **1**–**4** were not evaluated in this assay due to the limited sample weight.

## 3. Materials and Methods 

### 3.1. General Experimental Procedures

Silica gel (SiO_2_, 200−300 mesh, Qingdao Marine Chemical Inc., Qingdao, China) and Sephadex LH-20 (green herbs, Beijing, China) were used for column chromatography. Preparative HPLC was performed using a Shimadzu LC-20AT HPLC pump (Shimadzu Corporation, Nakagyo-ku, Kyoto, Japan) installed with an SPD-20A dual λ absorbance detector (Shimadzu Corporation, Nakagyo-ku, Kyoto, Japan) and a Shim-pack PRC-ODS HPLC column (250 mm × 20 mm, Shimadzu Corporation, Nakagyo-ku, Kyoto, Japan). 1D and 2D NMR spectra were recorded on Bruker Avance II 400 spectrometers (Bruker BioSpin AG, Industriestrasse 26, Fällanden, Switzerland) and a Varian Mercury-Plus 300 spectrometer (Varian Medical Systems In., Salt Lake City, UT, USA) in CDCl_3_ and acetone-*d*_6_. The chemical shifts are relative to the residual solvent signals (CDCl_3_: δ_H_ 7.260 and δ_C_ 77.160 and acetone-*d*_6_: δ_H_ 2.050 and δ_C_ 29.840). The high-resolution ESI-MS spectra was obtained with Shimadzu LCMS-IT-TOF (Shimadzu Corporation, Nakagyo-ku, Kyoto, Japan). UV spectra were recorded on a Shimadzu UV-Vis-NIR spectrophotometer (Shimadzu Corporation, Nakagyo-ku, Kyoto, Japan). IR spectra were recorded on a PerkinElmer Frontier FT-IR spectrophotometer (PerkinElmer Inc., Waltham, MA, USA). 

### 3.2. Fungal Strain and Culture Method

The marine fungus *Dichotomomyces sp*. L-8 was obtained from the inner tissue of the soft coral *Lobophytum crassum* collected from Hainan Sanya National Coral Reef Reserve, China. This fungal strain was maintained in 15% (v/v) glycerol aqueous solution at −80 °C. A voucher specimen was deposited in the School of Pharmaceutical Sciences, Sun Yat-sen University, Guangzhou, China. Analysis of the ITS rDNA (GenBank KF999816) by BLAST database screening provided a 100% match to *Dichotomomyces sp*. (AB734448).

The marine fungus, *Dichotomomyces sp*., was cultured in the medium which contained glucose 10 g/L, peptone 5 g/L, yeast extract 2 g/L, sea salt 22 g/L, H_2_O 1 L and pH 7.5. Fungal mycelia were cut and transferred aseptically to 1000 mL Erlenmeyer flasks each containing 400 mL sterilized liquid medium. The flasks were incubated at 28 °C for 40 days.

### 3.3. Extraction and Isolation

Seventy liters of liquid culture were filtered through cheesecloth. The culture broth was extracted three times with EtOAc and then was concentrated by low-temperature rotary evaporation to afford a crude extract (20 g).

The extract (20 g) was chromatographed on a silica gel column (diameter: 8 cm, length: 70 cm, silica gel, 200 g) with a gradient of petroleum ether-EtOAc (100:0–0:100, v/v) followed by EtOAc–MeOH (100:0–0:100, v/v) to yield seven fractions (Fr. 1–Fr. 7). Fr. 3 was purified by silica gel column using a step gradient elution with CHCl_2_–acetone (10:0–0:10, v/v) to get 10 subfractions (Fr. 3-1–Fr. 3-10) after pooling the similar fractions as monitored by TLC. Fr. 3–5 was chromatographed on Sephadex LH-20 (MeOH), then purified with preparative HPLC (MeOH–H_2_O, 60:40, v/v) to obtain compound **5** (5 mg) and **6** (4 mg). Fraction 4 was chromatographed on ODS (MeOH–H_2_O, 20:80–100:0, v/v), then on Sephadex LH-20 with MeOH as the eluent followed by preparative HPLC (MeOH–H_2_O, 63:37, v/v) to afford compound **3** (0.9 mg). Meanwhile, compound **4** (0.9 mg) was crystallized from Fr. 4-4. Fraction 5 was separated as six subfractions (Fr. 5-1–Fr. 5-6) with ODS using a gradient elution MeOH–H_2_O (20:80–100:0, v/v). Fr. 5-3 and Fr. 5-4 were further purified by Sephadex LH-20 (MeOH) and then by preparative HPLC (MeOH–H_2_O, 56:44, v/v; MeOH–H_2_O, 59:41, v/v), respectively, to acquire compound **1** (0.7 mg) and **2** (0.9 mg).

### 3.4. Characterization of Compounds ***1*** and ***2***

Dichotone A (**1**). White amorphous powder, UV (MeOH)*λ*_max_ (log ε): 235 (4.45), 201 (4.27); IR (KBr) *ν*_max_ 3392, 2965, 2926, 1714, 1670, 1262, 1155, 1094, 1048, 806 cm^−1^. ^1^H and ^13^C-NMR data, see Table 1; HRESIMS [M − H]^−^ *m*/*z* 241.1237 (calcd. for C_16_H_17_O_2_, 241.1234).

Dichotone B (**2**). White amorphous powder, UV (MeOH)*λ*_max_ (log ε): 225 (3.95), 200 (4.22); IR (KBr) *ν*_max_ 3436, 2962, 2925, 1714, 1513, 1262, 1097, 1084, 1036, 804 cm^−1^. ^1^H and ^13^C-NMR data, see Table 1; HRESIMS [M − H]^−^ *m*/*z* 269.1176 (calcd. for C_17_H_17_O_3_, 269.1183).

### 3.5. Cytotoxicity Assay

In vitro cytotoxicities of compounds **5** and **6** were carried out with human cancer cells MDA-MB-435 and Calu3 and evaluated by means of the colorimetric MTT assay. MDA-MB-435 and calu3 were seeded into 96-well plates each 100 μL/well at a density of 1 × 10^4^ cells per well and incubated at 37 °C for 24 h. Then the compounds were added to the cultures at various concentrations (0.125–50 μM). Then, 48 h later, 20 microliters MTT reagent (Genview, Houston, TX, USA) dissolved in phosphate-buffered saline (PBS) (pH 7.4) at a concentration of 5 mg·mL^−1^ were added into each well, and the cells were incubated at 37 °C for additional 4 h. The MTT-formazan crystals were formed and then dissolved in 150 mL DMSO (Sangon Biotech, Shanghai, China). The absorbance was recorded at 570 nm with a reference wavelength of 630 nm using a microplate reader. The half maximal inhibitory concentration (IC_50_) was calculated by Bliss’s software [18], and the data was calculated by SPSS.

## 4. Conclusions

In summary, two new compounds, dichotones A and B (**1** and **2**), and four known compounds, dichotocejpin C (**3**), bis-*N*-norgliovictin (**4**), bassiatin (**5**) and (3*R*,6*R*)-bassiatin (**6**), were obtained from the fungus *Dichotomomyces sp*. The compound (3*R*,6*R*)-bassiatin (**6**) is the epimer of bassiatin (**5**) at C-3. Compound **6** displayed notable cytotoxic activities against the human breast cancer cell line MDA-MB-435 and the human lung cancer cell line Calu3; however, compound **5** showed no cytotoxicity, which indicated that the 3*R*-configuration is essential for the cytotoxicity of compound **6**. It was supposed that only the 3*R*-configuration can specifically bind to the target molecule, such as protein kinase in signaling pathways*.* The absolute stereochemistry of natural products significantly influences their bioactivity due to the distinction in their recognition ability and binding force with the target organism. For example, the blocking action of the β receptor for *S*-(−)-propranolol was about 100-fold stronger than that of *R*-(+)-propranolol, while *R*-(+)-propranolol had an inhibitory effect on sodium channel. Therefore, the absolute configuration has become a critical issue in the drug development. Further investigation on the anticancer mechanism is in progress, which will facilitate clarifying the stereo-selectivity of (3*R*,6*R*)-bassiatin (**6**) on anticancer activity.

## Figures and Tables

**Table 1:** ^1^H- and ^13^C-NMR data of compounds **1** and **2** at 400/100 MHz, respectively, δ in ppm.

Pos.	1 ^a^		2 ^a^	
	δ_C_, Type	δ_H_, mult. (*J* in Hz)	δ_C_, Type	δ_H_, mult. (*J* in Hz)
1	149.9, C		126.4, C	
2	114.1, C		130.8, CH	7.02, d (9.2)
3	131.7, C		115.7, CH	6.77, d (8.4)
4	124.6, CH	7.61, s	154.7, C	
5	131.5, C		115.7, CH	6.77, d (8.4)
6	132.0, C		130.8, CH	7.02, d (9.2)
7	128.6, CH	7.49, s	49.6, CH_2_	3.57, s
8	125.6, C		208.3, CO	
9	126.2, CH	7.39, s	43.7, CH_2_	2.71, td (6.8, 1.6)
10	128.8, C		29.1, CH_2_	2.80, td (6.8, 1.6)
11	10.8, CH_3_	2.50, s	133.1, C	
12	50.1, CH_2_	3.87, s	129.4, CH	7.04, d (8.8)
13	207.2, CO		114.0, CH	6.79, d (8.8)
14	29.4, CH_3_	2.16, s	158.0, C	
15	19.9, CH_3_	2.34, s	114.0, CH	6.79, d (8.8)
16	17.1, CH_3_	2.41, s	129.4, CH	7.04, d (8.8)
17			55.4, OCH_3_	3.77, s
1-OH		4.87, s		
5-OH				4.83, s

^a^ Measured in CDCl_3_.

## Supplementary Materials

Supplementary materials are available online.
