# Supplementary material for: Secondary Metabolites from the Marine-Derived Fungus Dichotomomyces sp. L-8 and Their Cytotoxic Activity
Source: Molecules. 2017 Mar 11;22(3):444. doi: 10.3390/molecules22030444 (PMC6155177; doi:10.3390/molecules22030444)

Supporting Information

Secondary Metabolites from the Marine-Derived Fungus *Dichotomomyces* sp. L-8 and Their Cytotoxic Activity

Li-Hong Huang [1,2], Yan-Xiu Chen [1,2], Jian-Chen Yu [3], Jie Yuan [3], Hou-Jin Li [4], Wen-Zhe Ma [5], Ramida Watanapokasin [6], Kun-Chao Hu [1,2], Shah Iram Niaz [7], De-Po Yang [1,2] and Wen-Jian Lan [1,2,*]

[1] School of Pharmaceutical Sciences, Sun Yat-sen University, Guangzhou 510006, China; huanglh23@mail2.sysu.edu.cn (L.H.H.); chenyx239@mail2.sysu.edu.cn (Y.X C.); Hukch@mail2.sysu.edu.cn (K.C.H.); lssydp@mail.sysu.edu.cn (D.P.Y.)

[2] Guangdong Technology Research Center for Advanced Chinese Medicine, Guangzhou 510006, China

[3] Zhongshan School of Medicine, Sun Yat-sen University, 74 Zhongshan Road II, Guangzhou 510080, China; lisana121406@163.com (J.-C.Y.); yuanjie@mail.sysu.edu.cn (J.Y.)

[4] School of Chemistry, Sun Yat-sen University, Guangzhou 510275, China; ceslhj@mail.sysu.edu.cn

[5] State Key Laboratory of Quality Research in Chinese Medicine, Macau Institute for Applied Research in Medicine and Health, Macau University of Science and Technology, Avenida Wai Long, Taipa 519020, Macau (SAR), China; wzma@must.edu.mo

[6] Department of Biochemistry, Faculty of Medicine, Srinakharinwirot University, Bangkok 10110, Thailand; ramidawa@yahoo.com

[7] Institute of Chemical Sciences, Gomal University, D.I.Khan 29050, Pakistan; shah_iram2000@Yahoo.com

* Correspondence: lanwj@mail.sysu.edu.cn; Tel.: +86-020-3994-3042

List of Content

Supporting Information........1
Figure S1. HR-ESI-MS spectrum of dichotone A (**1**)........s1
Figure S2. ^{1}H NMR spectrum of dichotone A (**1**) in $CDCl_3$ (400MHz)........s2
Figure S3. ^{13}C NMR spectrum of dichotone A (**1**) in $CDCl_3$ (100MHz)........s3
Figure S4. DEPT 135 spectrum of dichotone A (**1**) in $CDCl_3$ (100MHz)........s4
Figure S5. HMQC spectrum of dichotone A (**1**) in $CDCl_3$........s5
Figure S6. ^{1}H-^{1}H COSY spectrum of dichotone A (**1**) in $CDCl_3$........s6
Figure S7. HMBC spectrum of dichotone A (**1**) in $CDCl_3$........s7
Figure S8. HR-ESI-MS spectrum of dichotone B (**2**)........s8
Figure S9. ^{1}H NMR spectrum of dichotone B (**2**) in $CDCl_3$ (400MHz)........s9
Figure S10. ^{13}C NMR spectrum of dichotone B (**2**) in $CDCl_3$ (100MHz)........s10
Figure S11. DEPT 135 spectrum of dichotone B (**2**) in $CDCl_3$ (100MHz)........s11
Figure S12. HMQC spectrum of dichotone B (**2**) in $CDCl_3$........s12
Figure S13. ^{1}H-^{1}H COSY spectrum of dichotone B (**2**) in $CDCl_3$........s13
Figure S14. HMBC spectrum of dichotone B (**2**) in $CDCl_3$........s14
Figure S15. ^{1}H NMR spectrum of dichotocejpin C (**3**) in acetone-d_6 (400MHz)........s15
Figure S16. ^{13}C NMR spectrum of dichotocejpin C (**3**) in acetone-d_6 (100MHz)........s16
Figure S17. ^{1}H NMR spectrum of bis-*N*-norgliovictin (**4**) in DMSO-d_6 (400MHz)........s17
Figure S18. ^{13}C NMR spectrum of bis-*N*-norgliovictin (**4**) in DMSO-d_6 (100MHz)........s18
Figure S19. ^{1}H NMR spectrum of bassiatin (**5**) in $CDCl_3$ (400MHz)........s19
Figure S20. ^{13}C NMR spectrum of bassiatin (**5**) in $CDCl_3$ (100MHz)........s20
Figure S21. ^{1}H NMR spectrum of 3*R*, 6*R*-bassiatin (**6**) in $CDCl_3$ (400MHz)........s21
Figure S22. ^{13}C NMR spectrum of 3*R*, 6*R*-bassiatin (**6**) in $CDCl_3$ (100MHz)........s22

Figure S1. HR-ESI-MS spectrum of dichotone A (**1**)

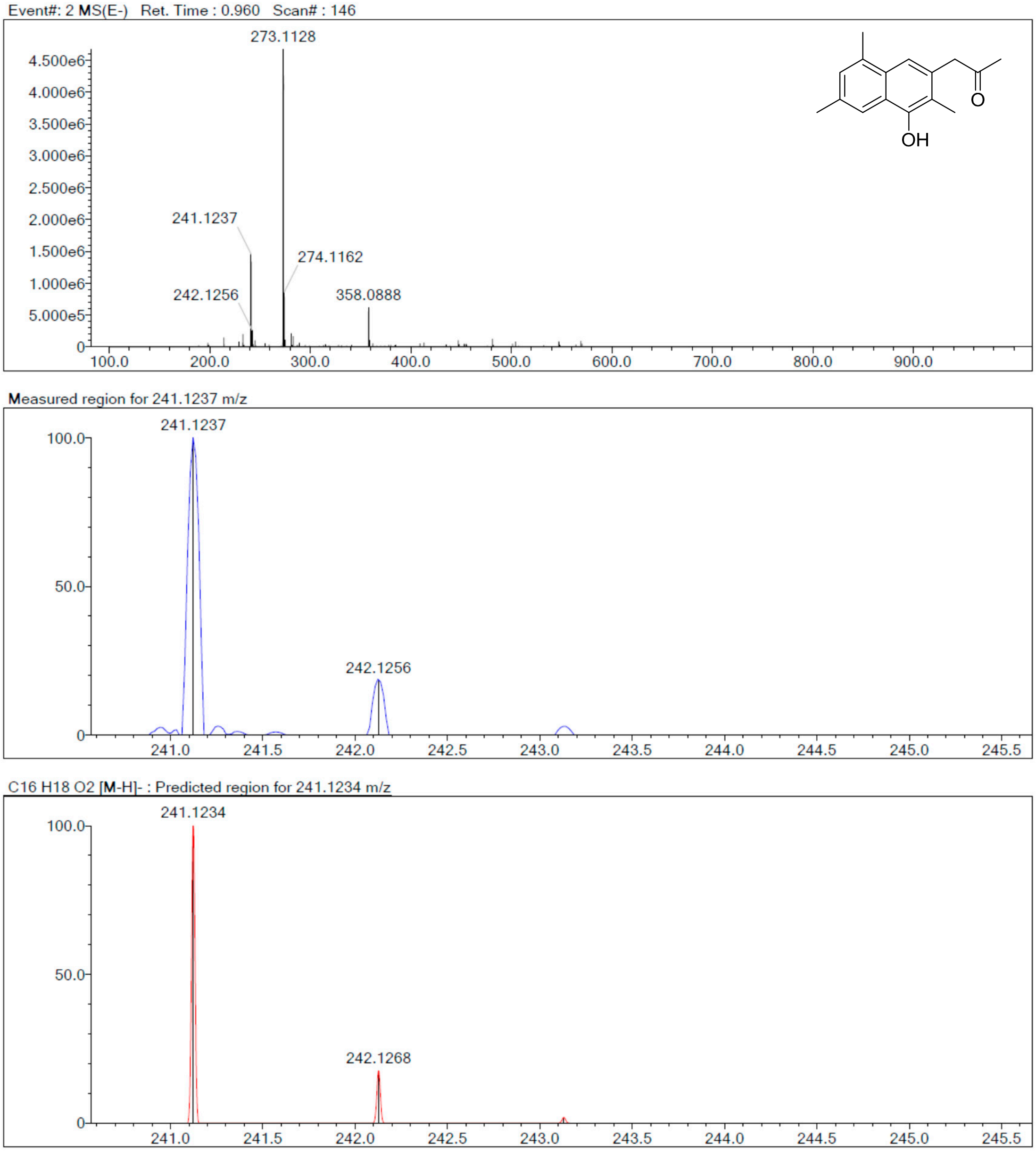

Rank	Score	Formula (M)	Ion	Meas. m/z	Pred. m/z	Df. (mDa)	Df. (ppm)	Iso	DBE
1	78.68	C16 H18 O2	[M-H]-	241.1237	241.1234	0.3	1.24	79.16	8.0

Figure S2. ^{1}H NMR spectrum of dichotone A (**1**) in $CDCl_3$ (400MHz)

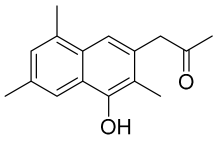

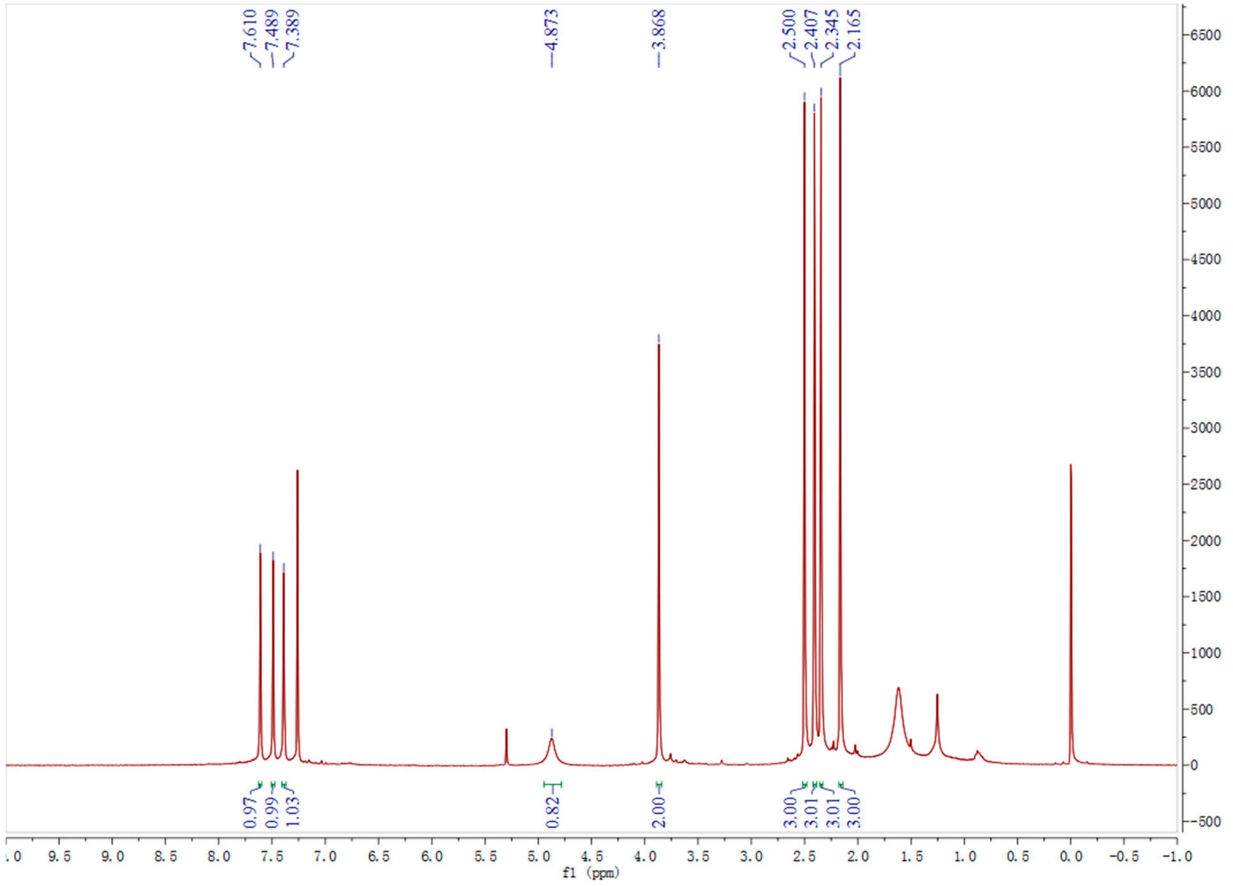

Figure S3. ^{13}C NMR spectrum of dichotone A (**1**) in $CDCl_3$ (100MHz)

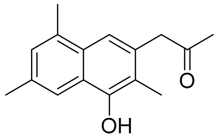

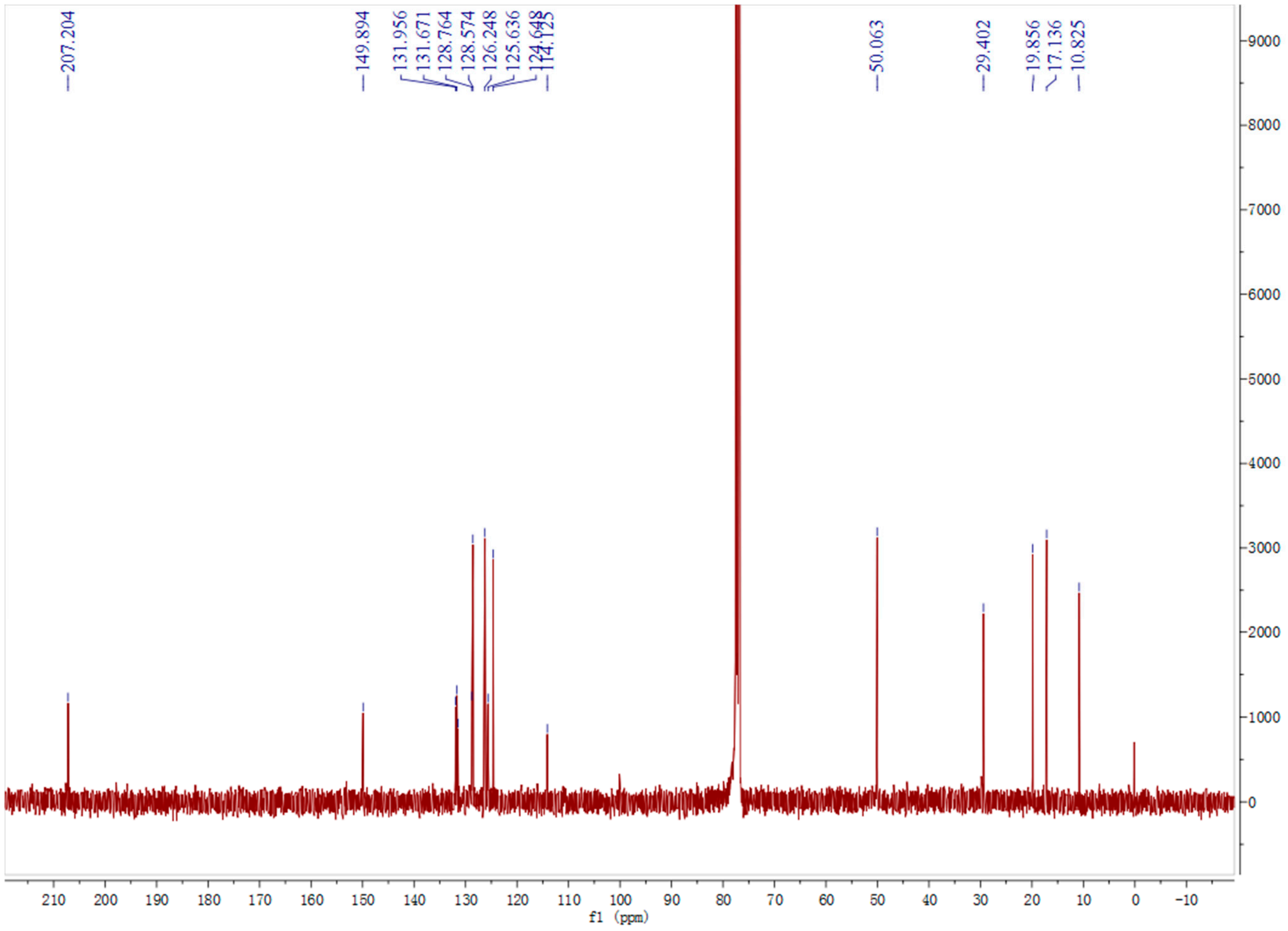

Figure S4. DEPT 135 spectrum of dichotone A (**1**) in $CDCl_3$ (100MHz)

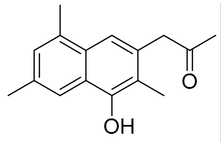

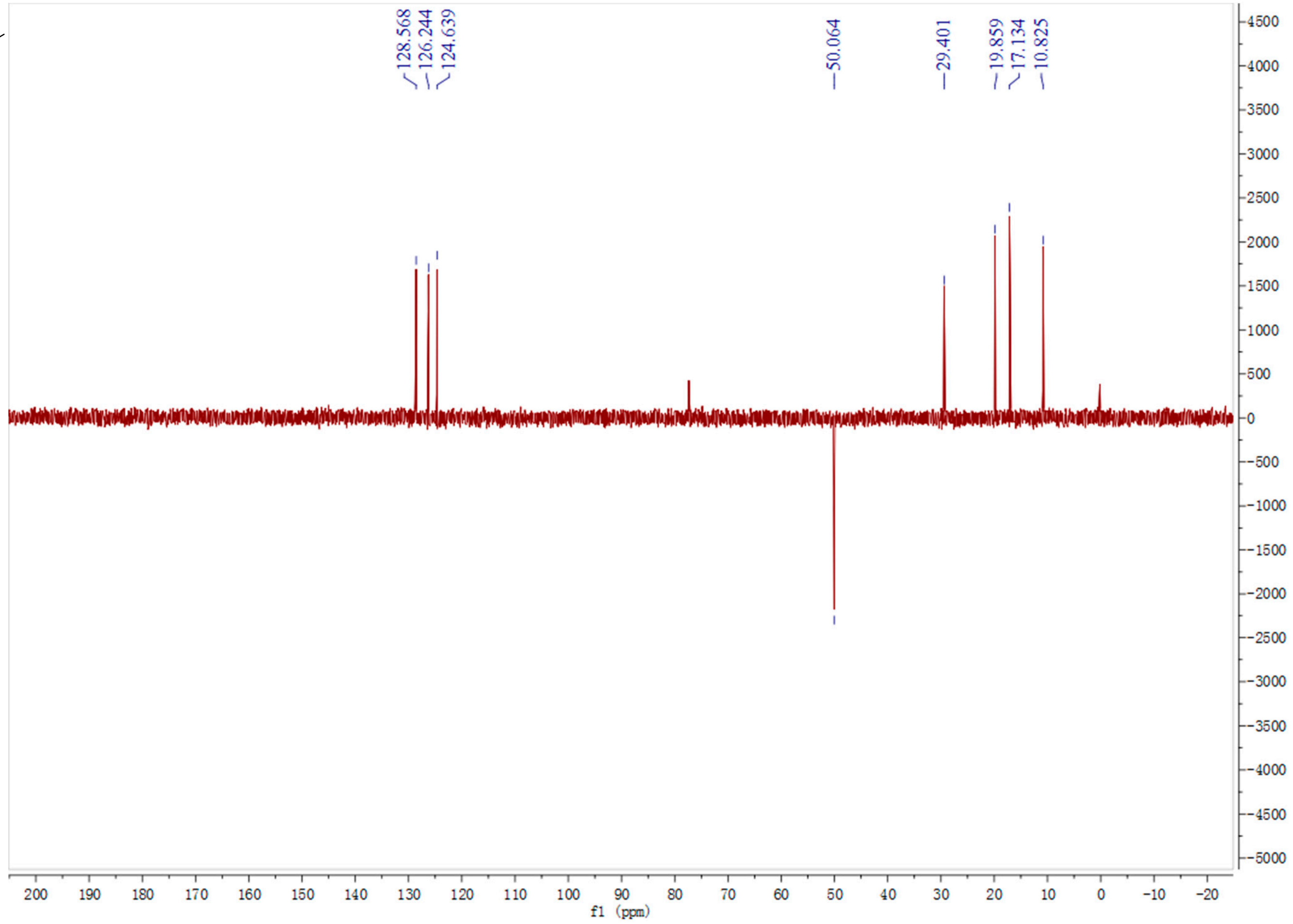

Figure S5. HMQC spectrum of dichotone A (**1**) in $CDCl_3$

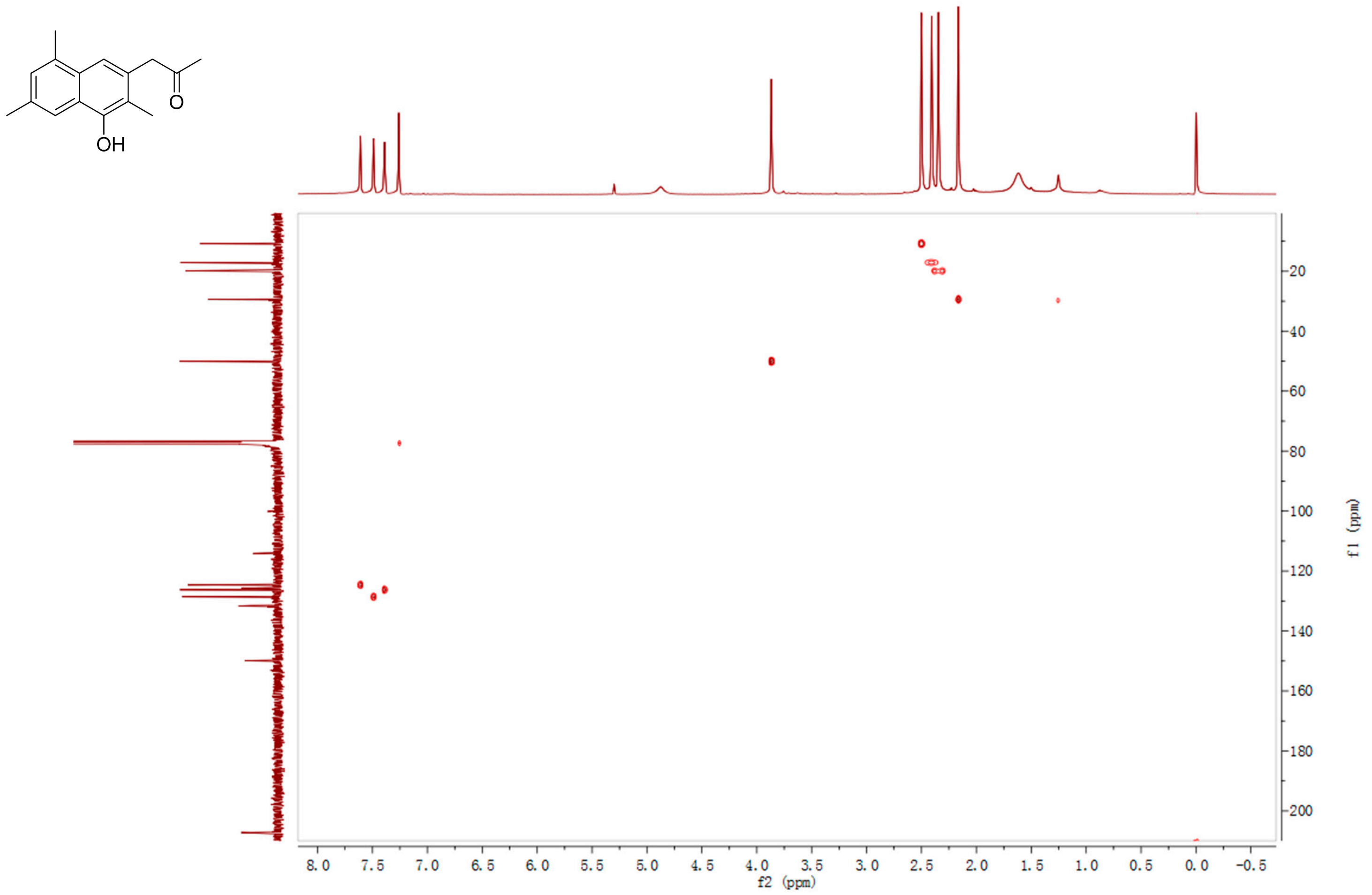

Figure S6. ^{1}H-^{1}H COSY spectrum of dichotone A (**1**) in $CDCl_3$

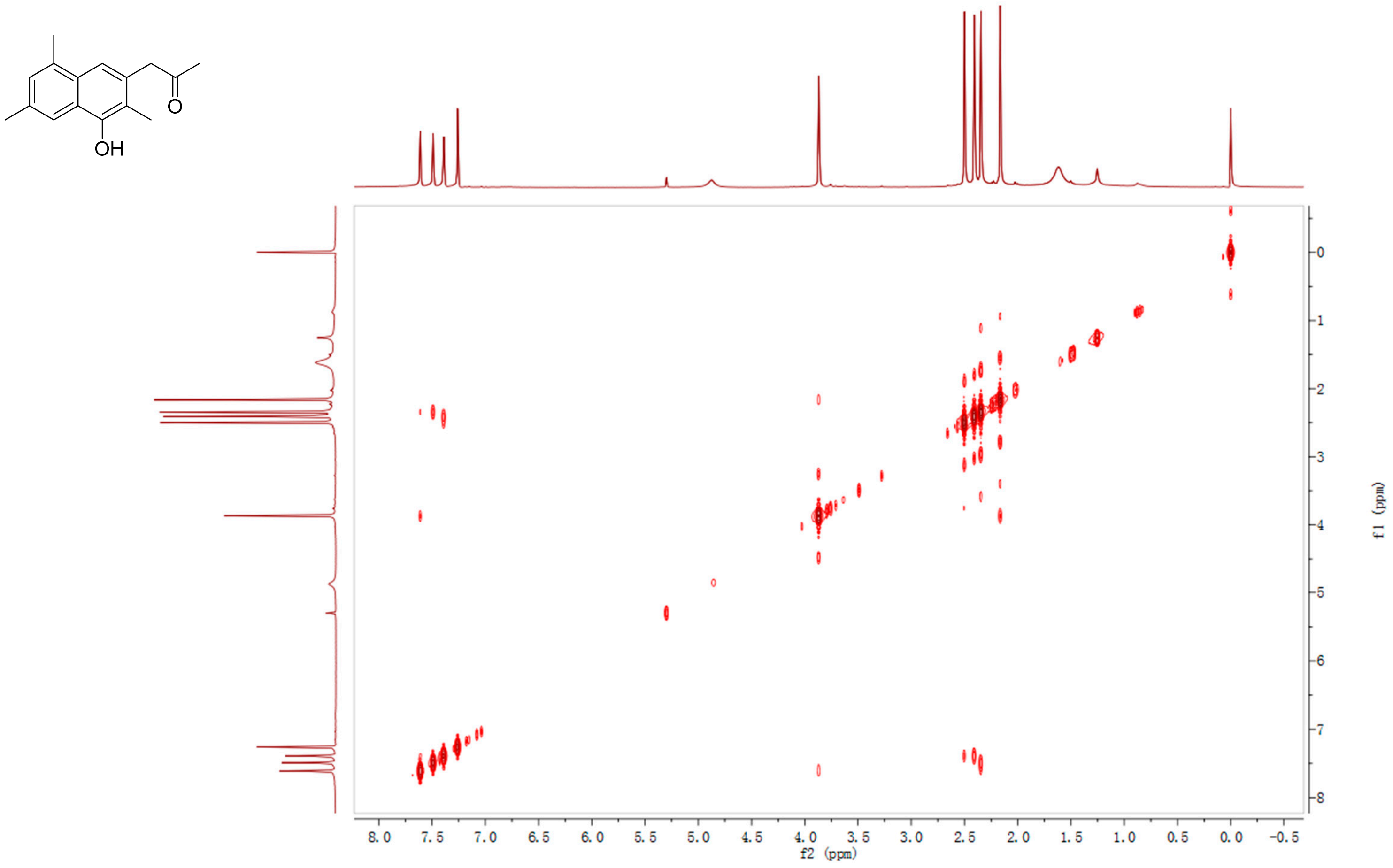

Figure S7. HMBC spectrum of dichotone A (**1**) in $CDCl_3$

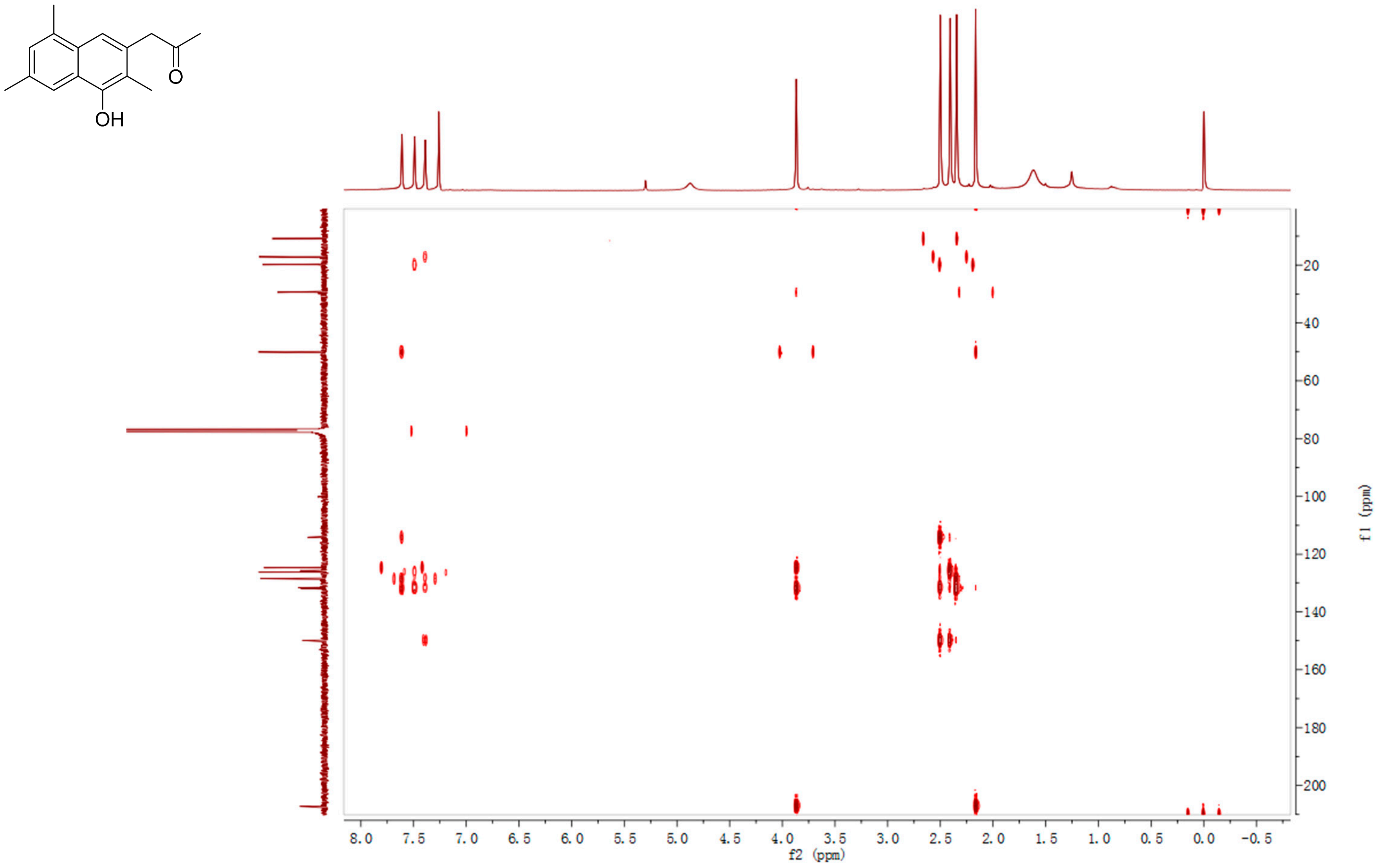

Figure S8. HR-ESI-MS spectrum of dichotone B (**2**)

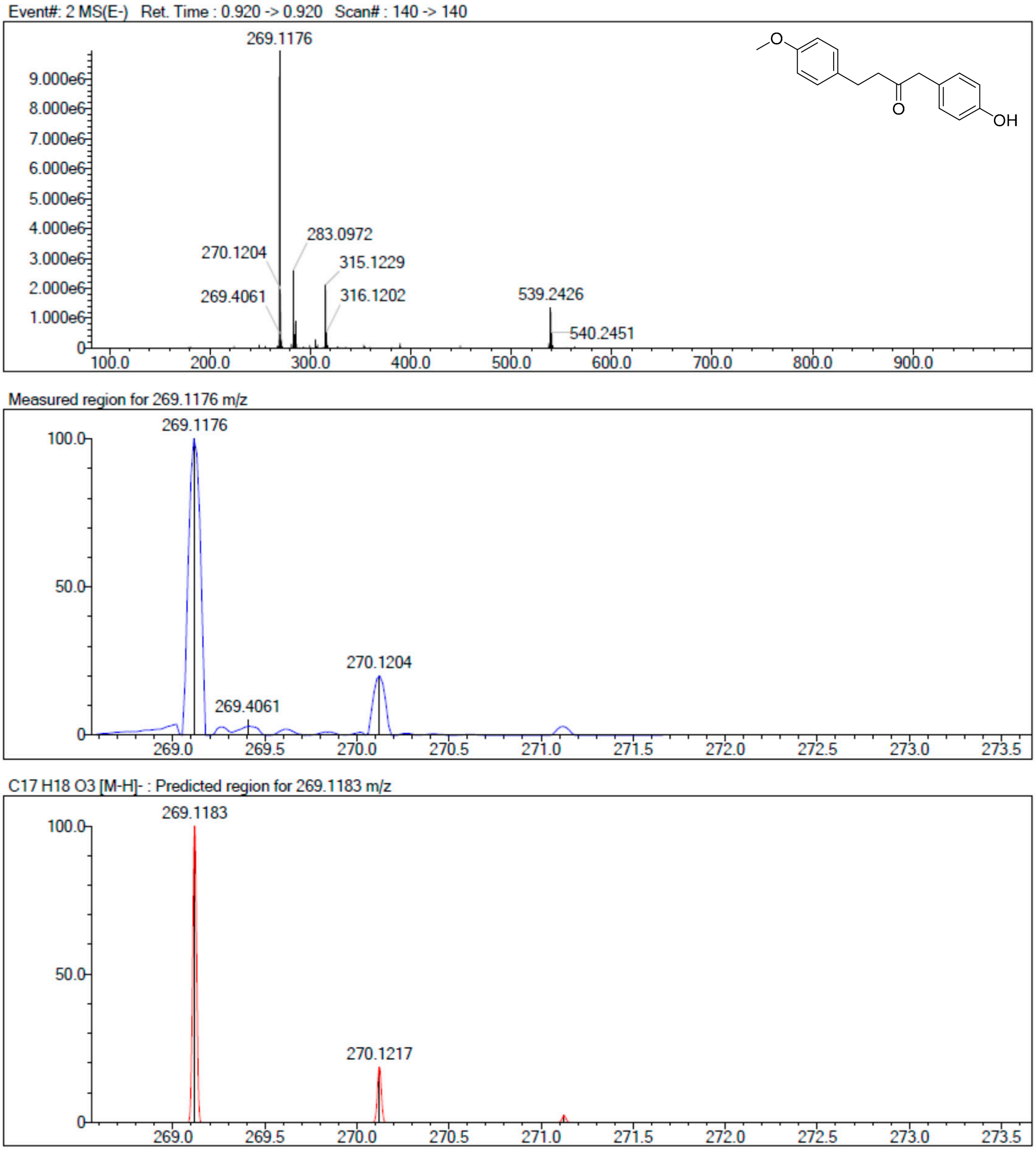

Rank	Score	Formula (M)	Ion	Meas. m/z	Pred. m/z	Df. (mDa)	Df. (ppm)	Iso	DBE
1	71.08	C17 H18 O3	[M-H]-	269.1176	269.1183	-0.7	-2.60	74.05	9.0

Figure S9. ^{1}H NMR spectrum of dichotone B (**2**) in $CDCl_3$ (400MHz)

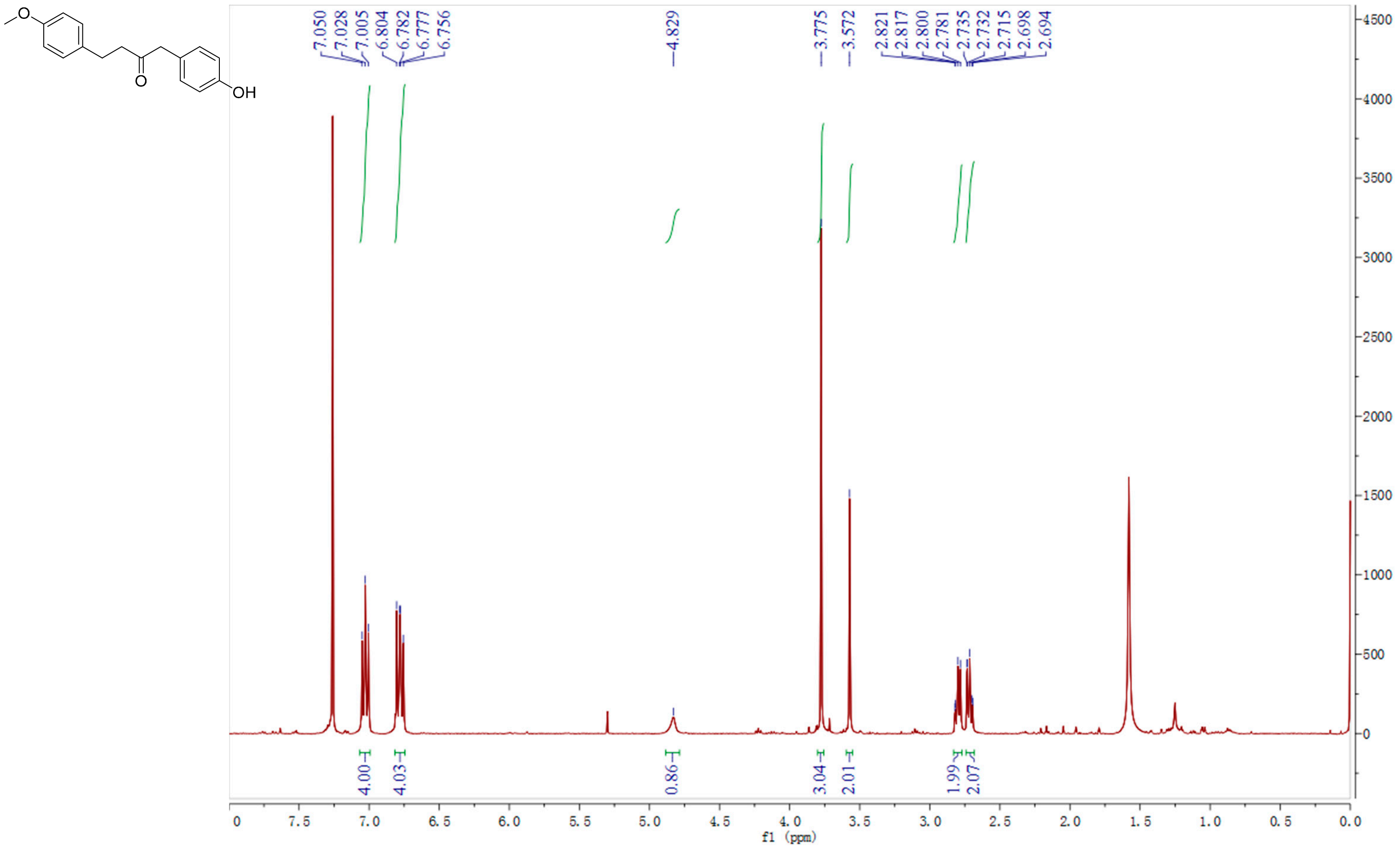

Figure S10. ^{13}C NMR spectrum of dichotone B (**2**) in $CDCl_3$ (100MHz)

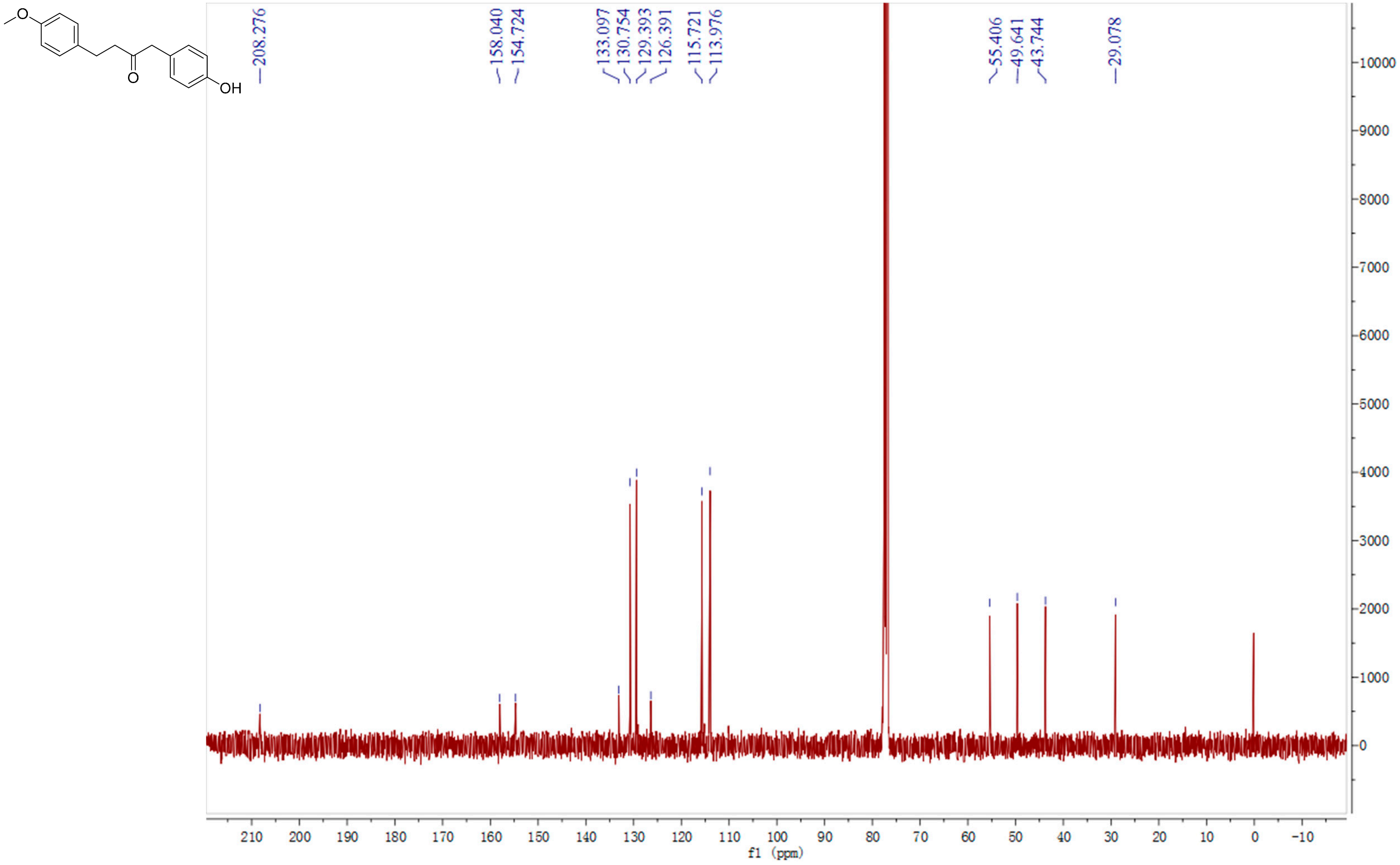

Figure S11. DEPT 135 spectrum of dichotone B (**2**) in $CDCl_3$ (100MHz)

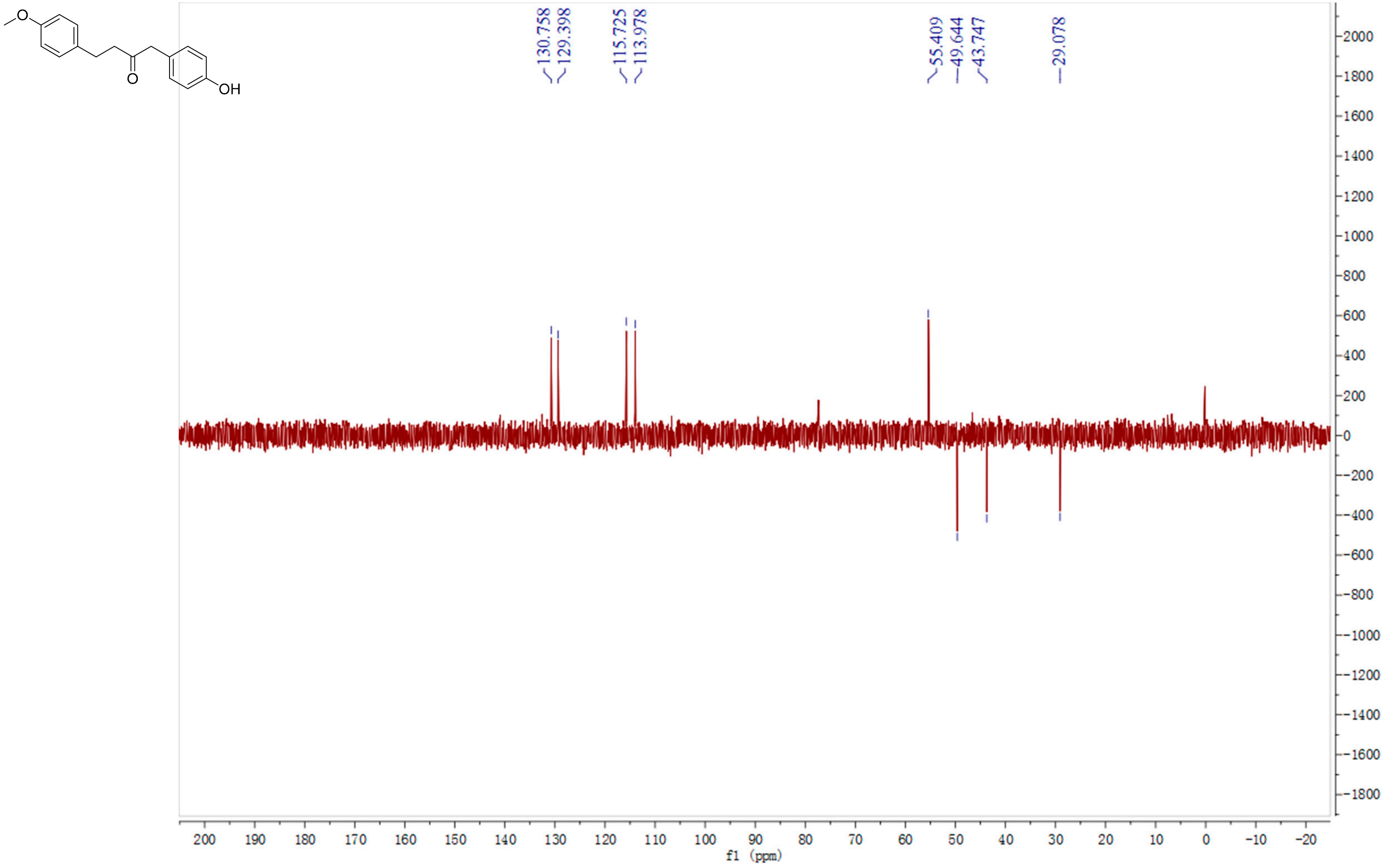

Figure S12. HMQC spectrum of dichotone B (**2**) in $CDCl_3$

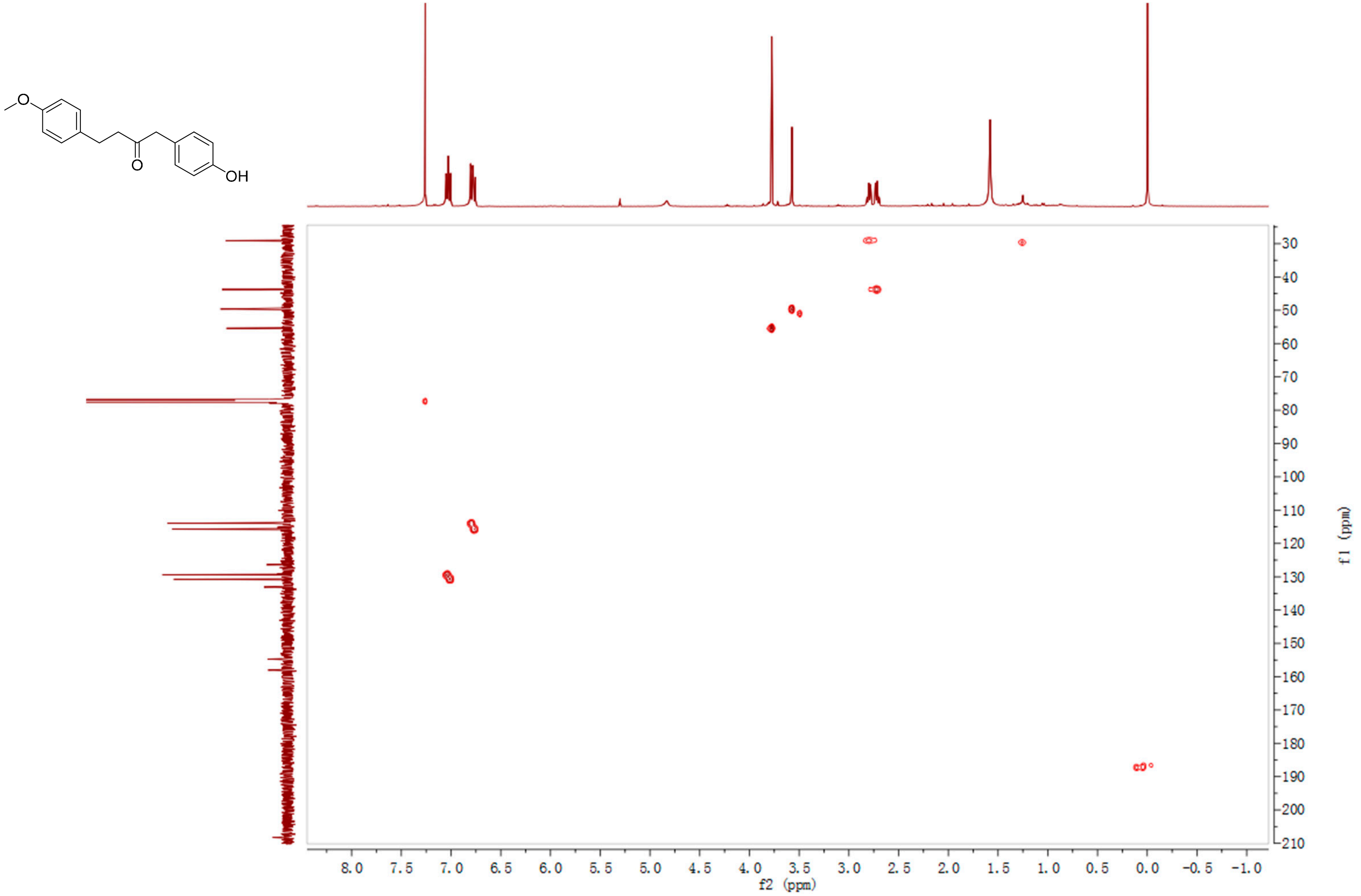

Figure S13. ^{1}H-^{1}H COSY spectrum of dichotone B (**2**) in $CDCl_3$

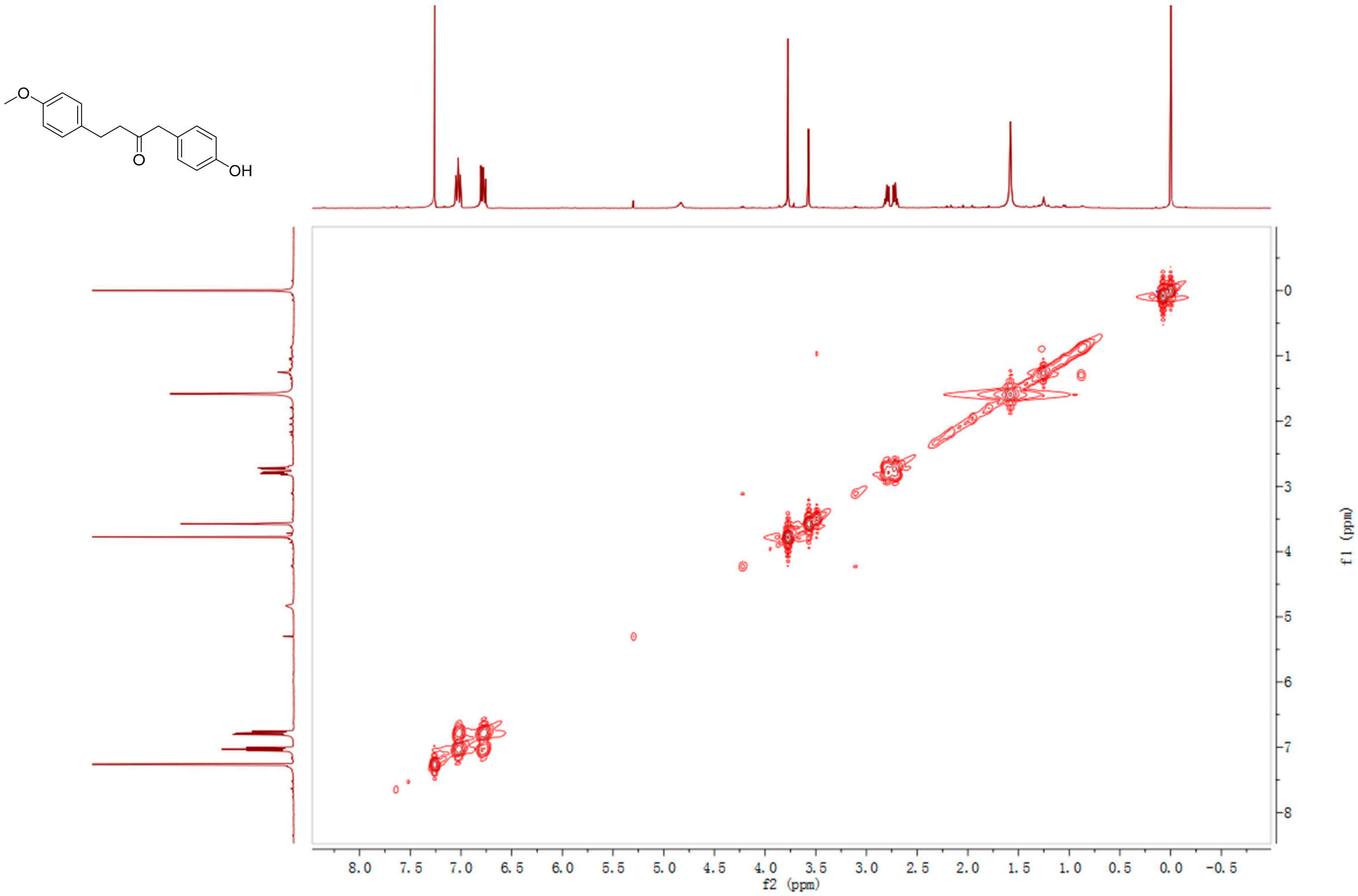

Figure S14. HMBC spectrum of dichotone B (**2**) in $CDCl_3$

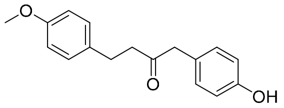

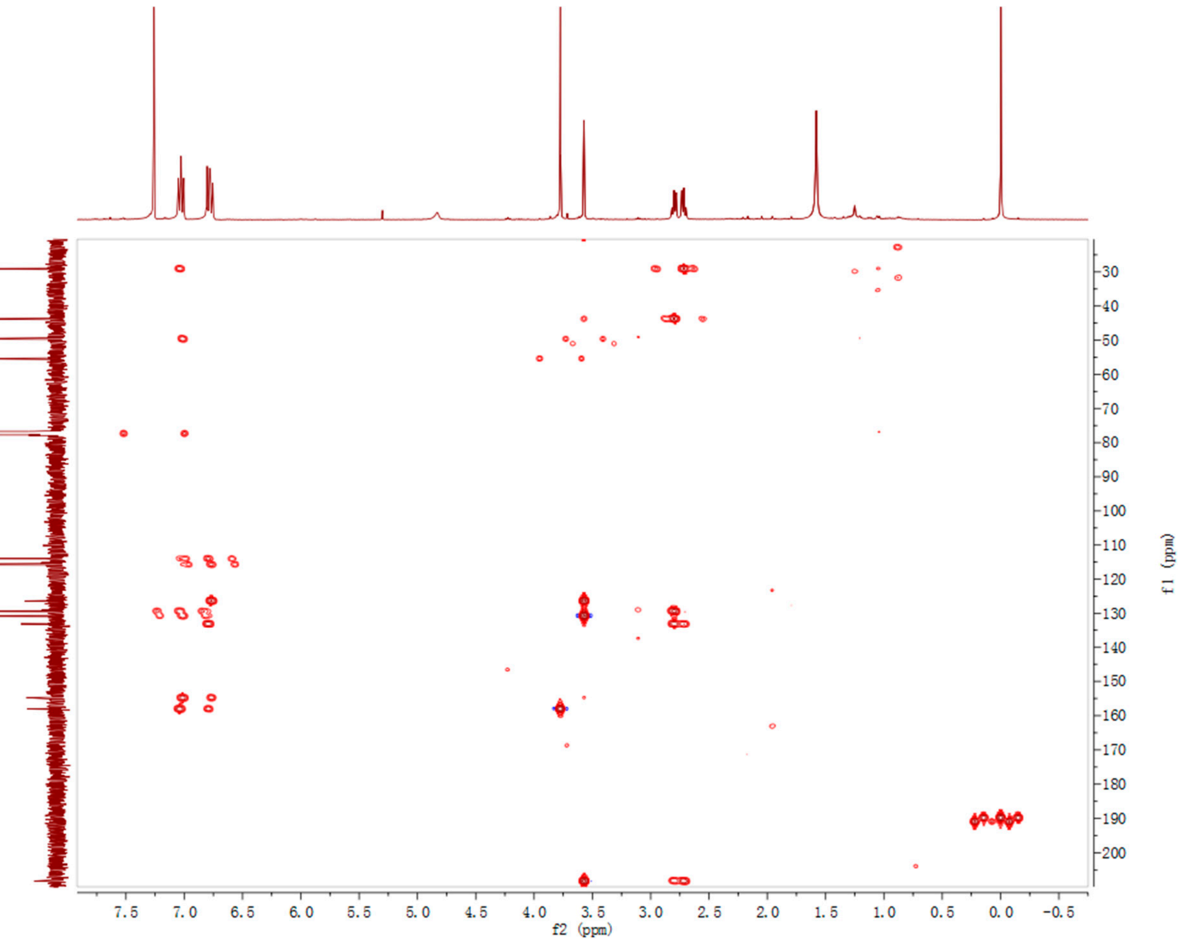

Figure S15. ^{1}H NMR spectrum of dichotocejpin C (**3**) in acetone-d_6 (400MHz)

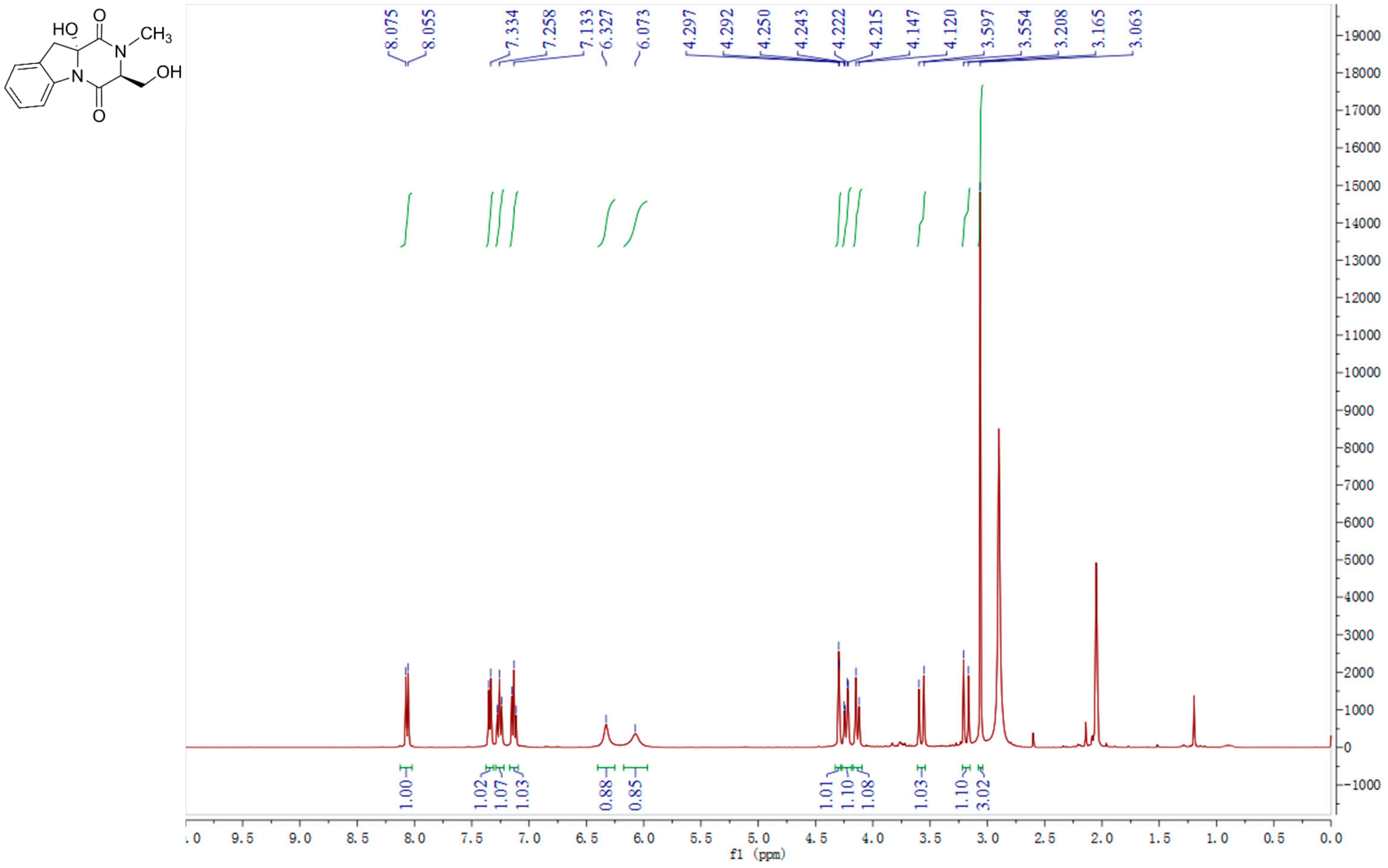

Figure S16. ^{13}C NMR spectrum of dichotocejpin C (**3**) in acetone-d_6 (100MHz)

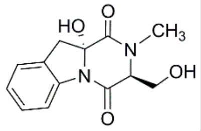

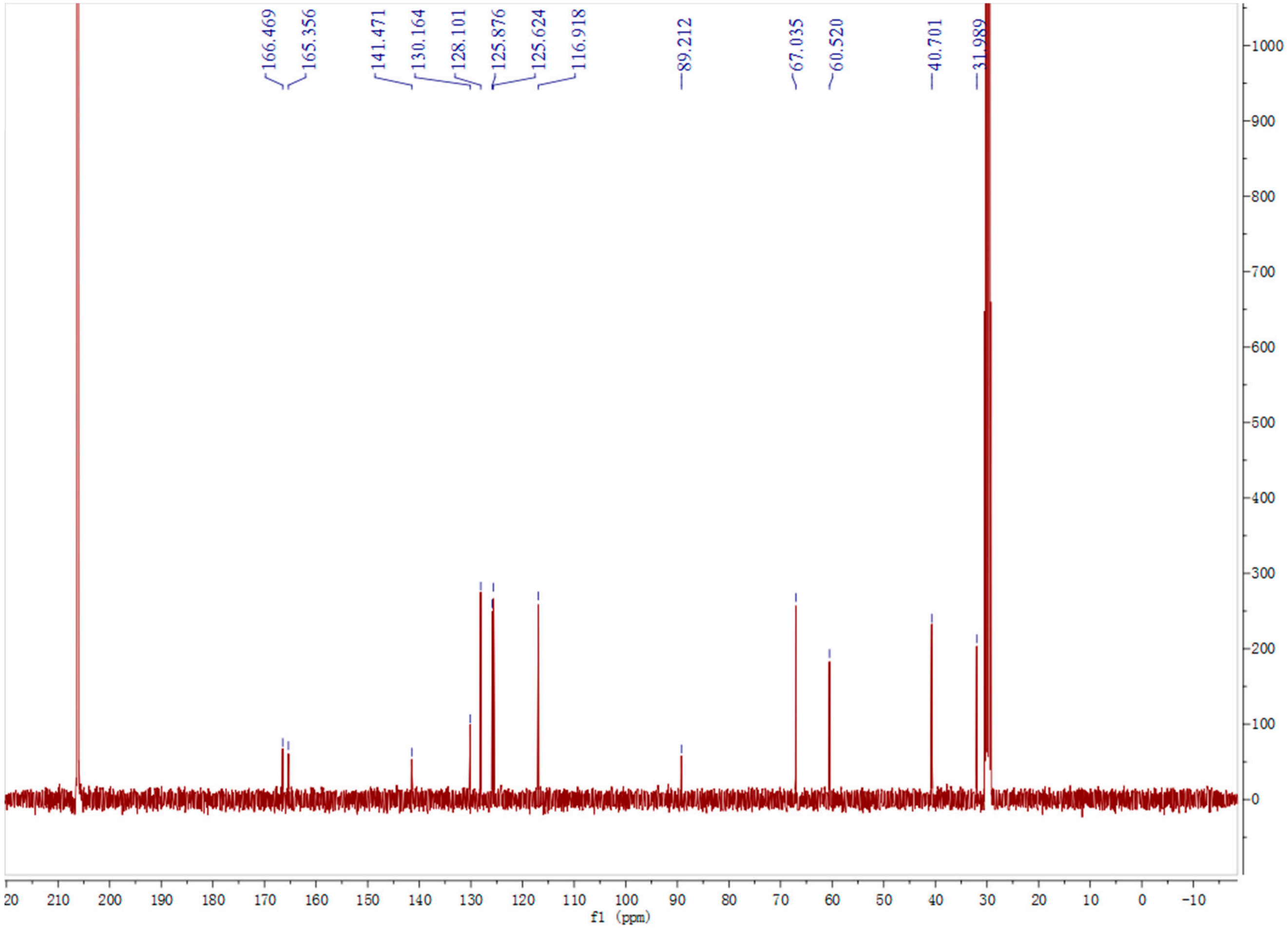

Figure S17. ^{1}H NMR spectrum of bis-*N*-norgliovictin (**4**) in DMSO-d_6 (400MHz)

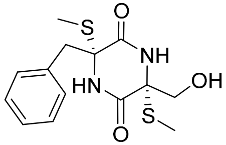

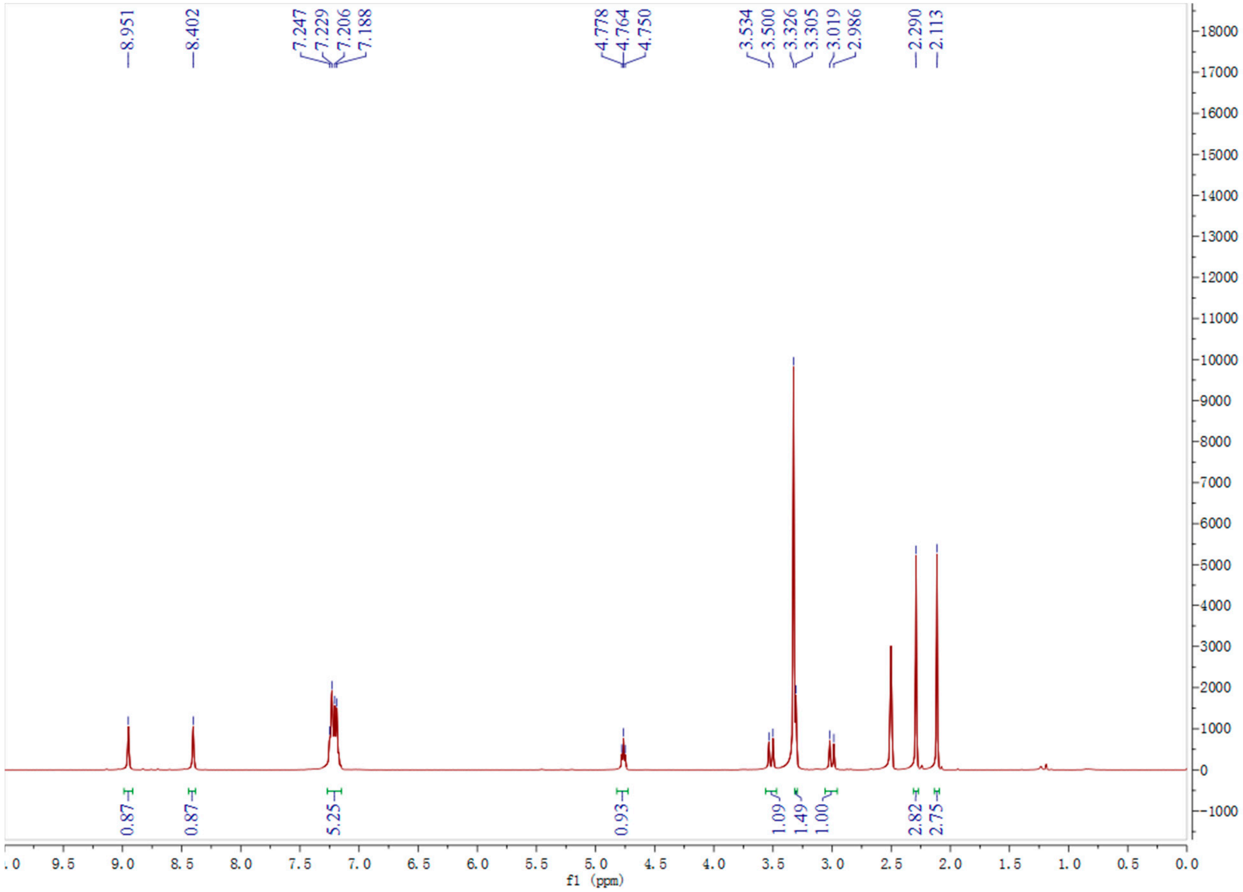

Figure S18. ^{13}C NMR spectrum of bis-*N*-norgliovictin (**4**) in DMSO-d_6 (100MHz)

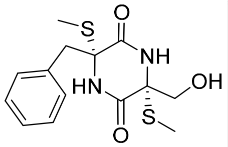

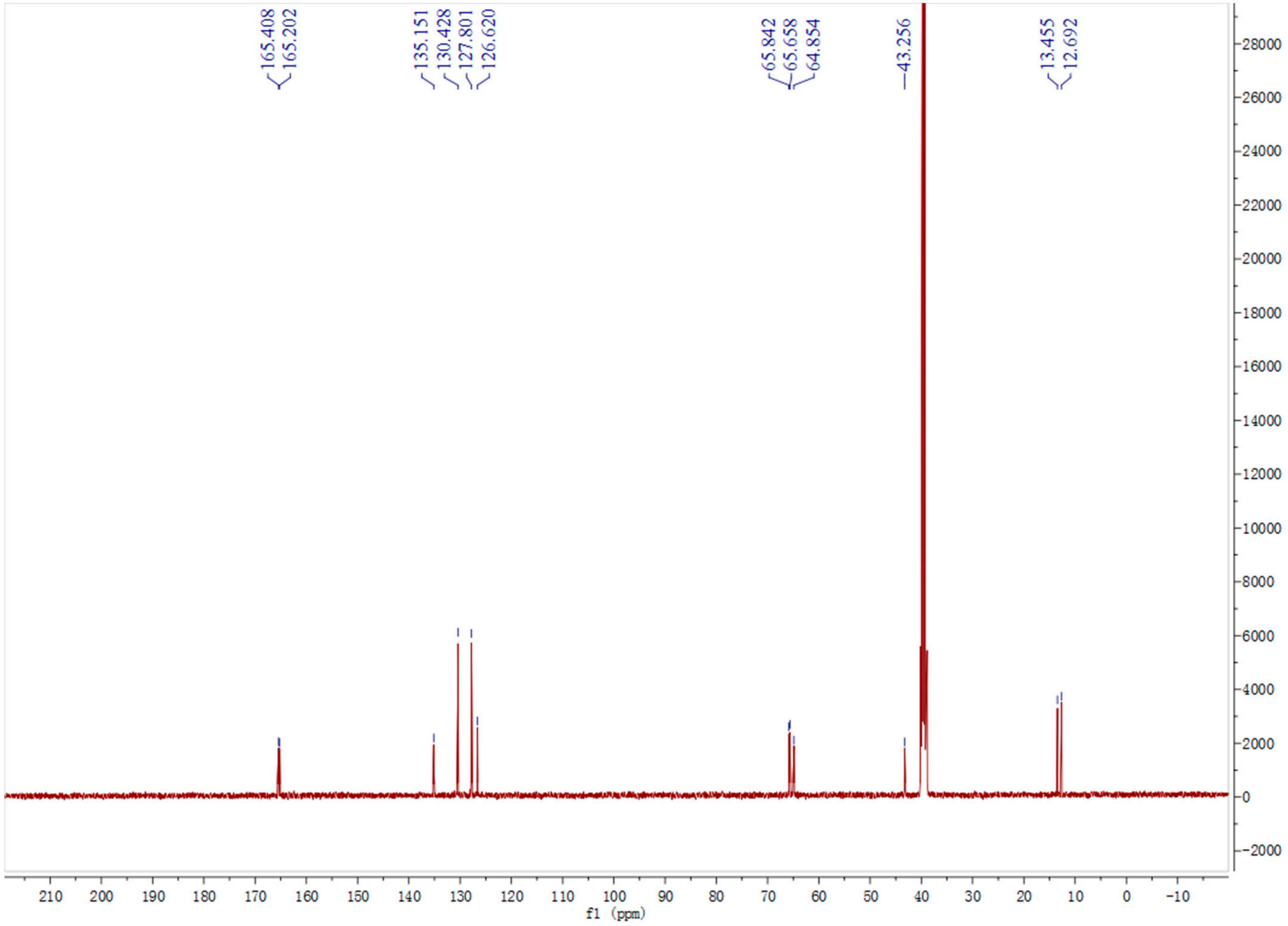

Figure S19. ^{1}H NMR spectrum of bassiatin (**5**) in $CDCl_3$ (400MHz)

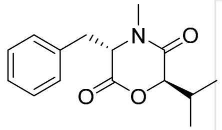

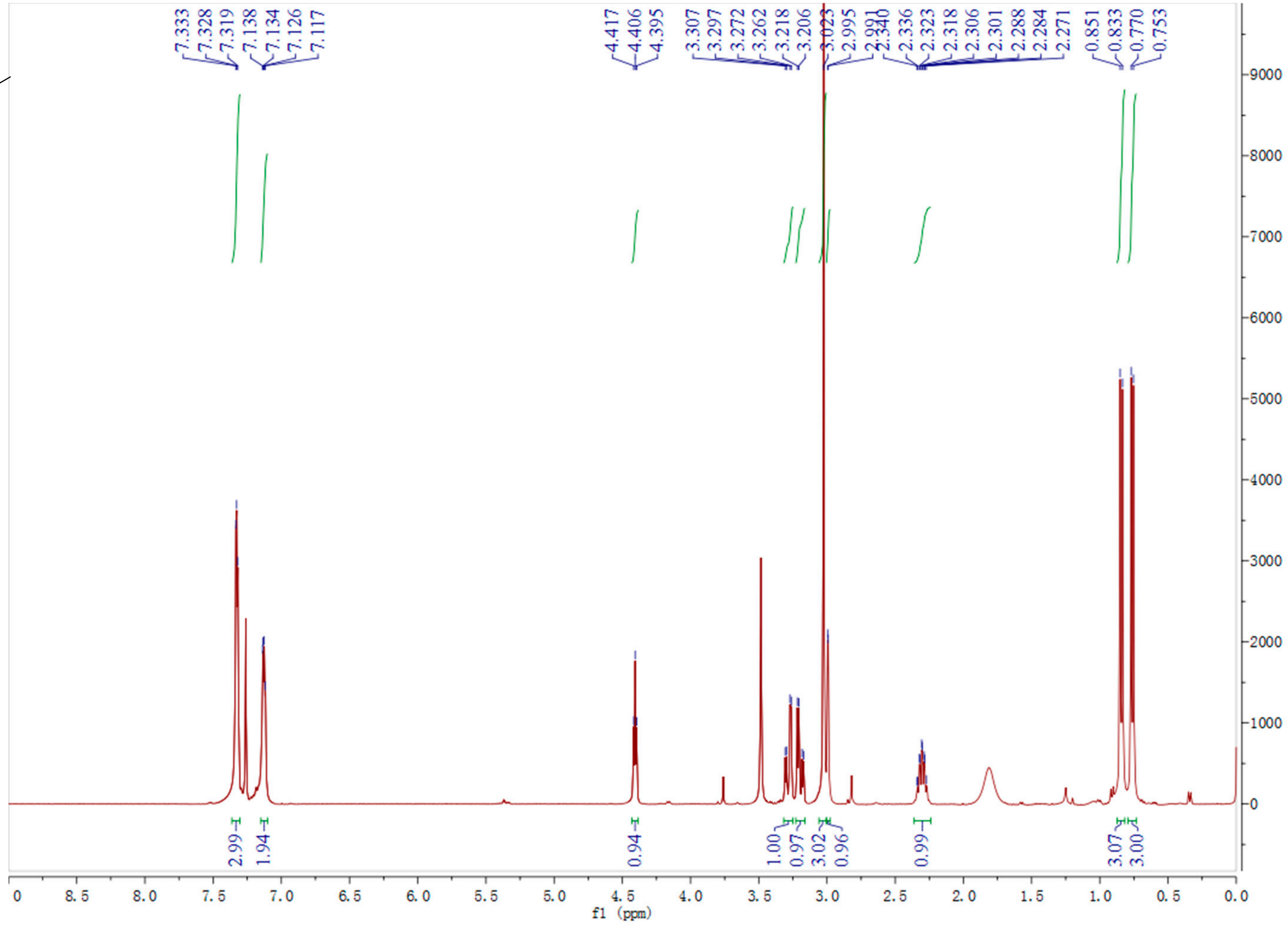

Figure S20. ^{13}C NMR spectrum of bassiatin (**5**) in $CDCl_3$ (100MHz)

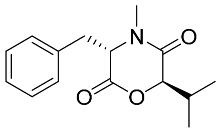

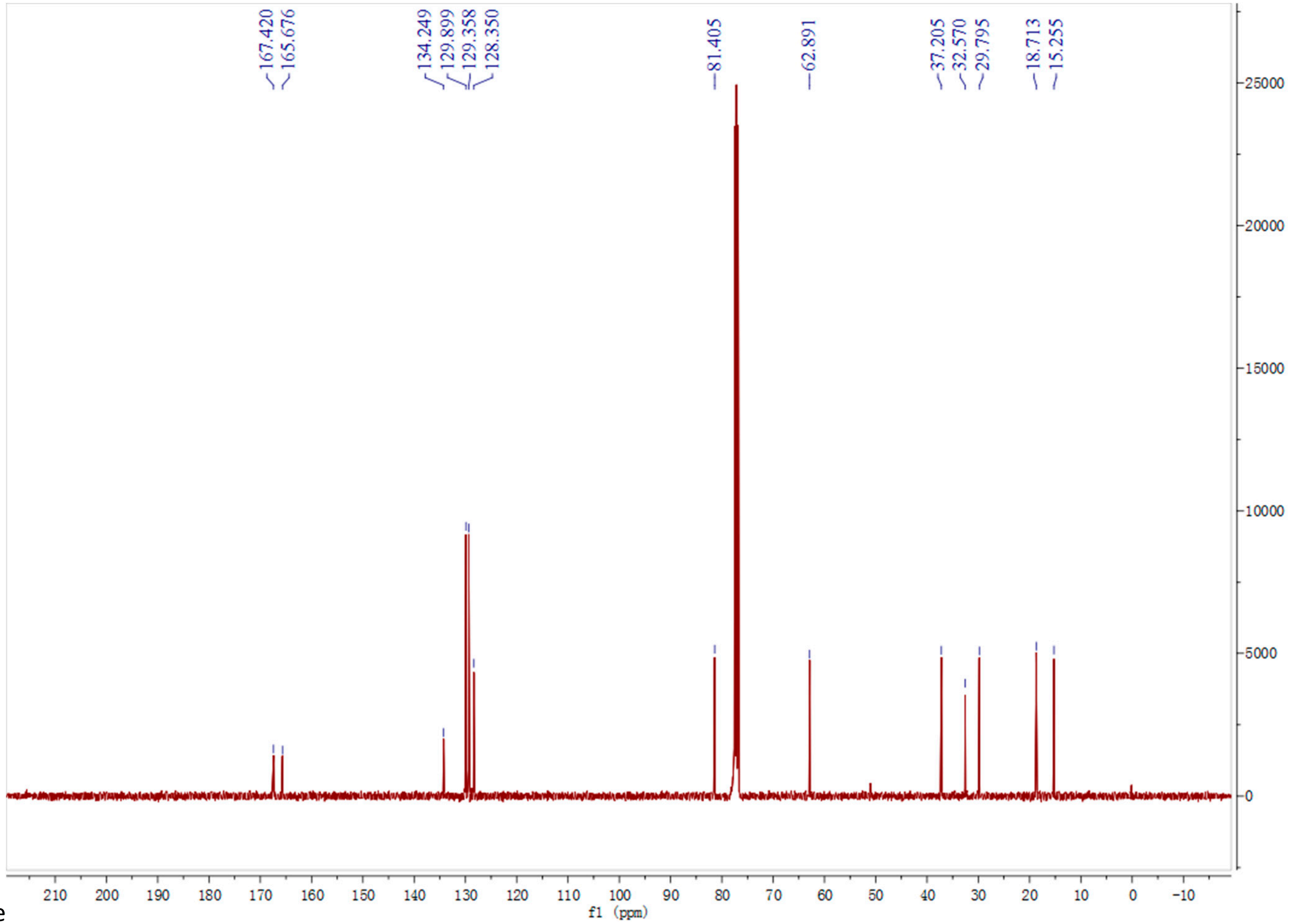

e

Figure S21. ^{1}H NMR spectrum of 3*R*, 6*R*-bassiatin (**6**) in $CDCl_3$ (400MHz)

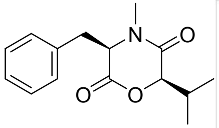

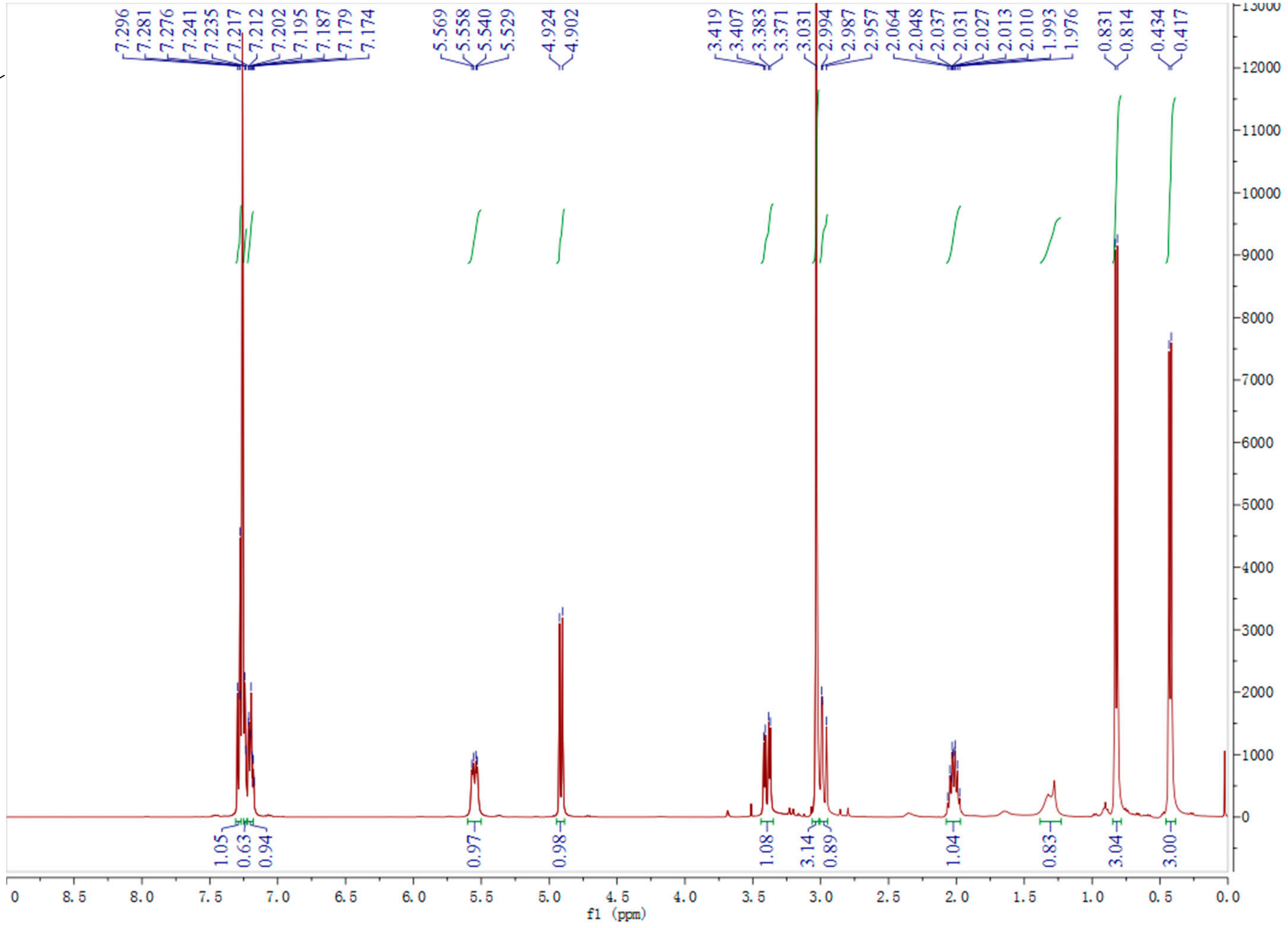

Figure S22. ^{13}C NMR spectrum of 3*R*, 6*R*-bassiatin (**6**) in $CDCl_3$ (100MHz)

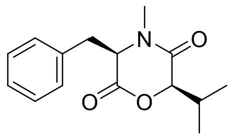

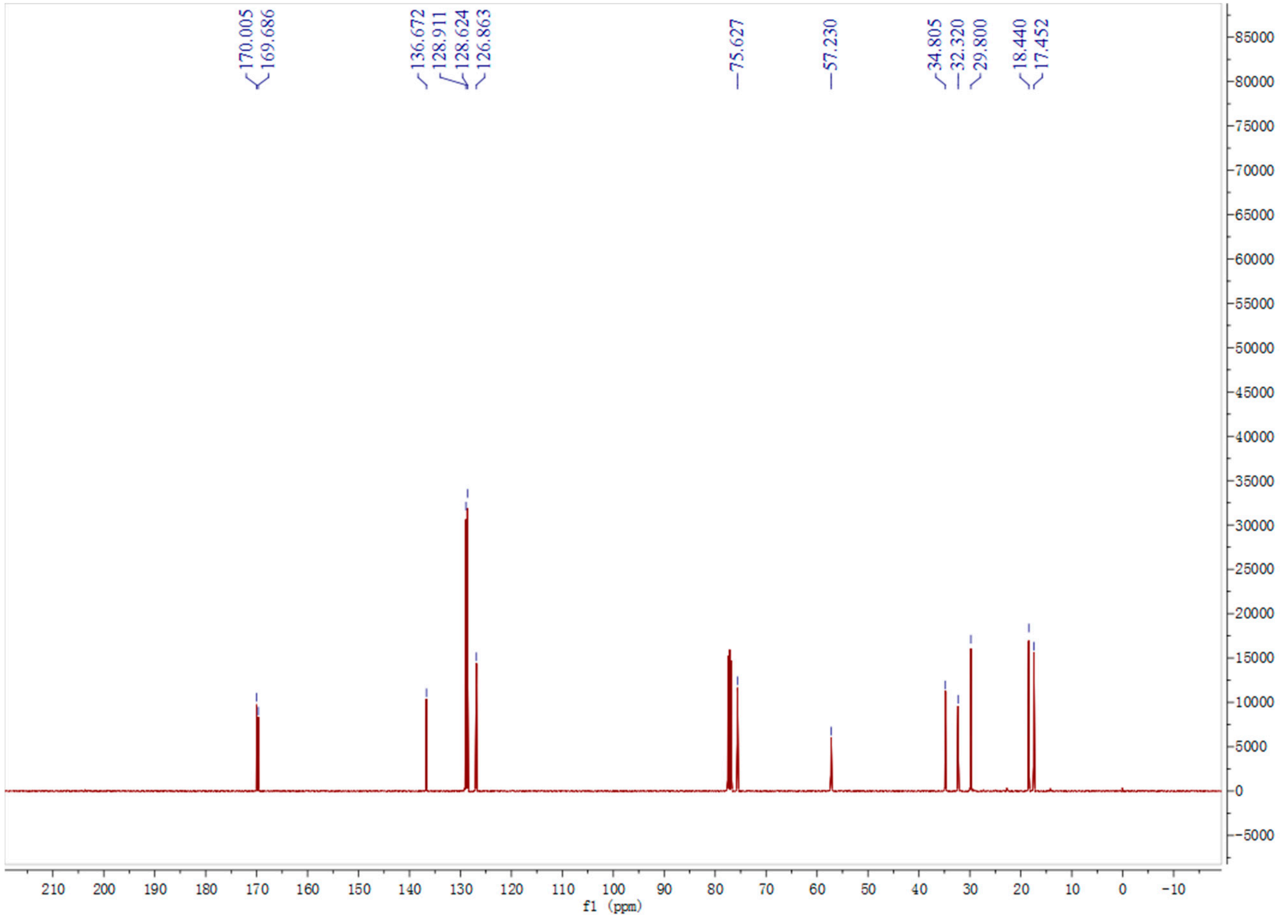

Supplement: Supplementary file 1 [file molecules-22-00444-s001.pdf]
